# Breathing exercises as adjunct therapy in adult asthma: a systematic review

**DOI:** 10.12688/f1000research.180230.1

**Published:** 2026-06-23

**Authors:** Astrid Crespo-Lessmann, Eduardo Vélez-Segovia, Luis Paz, Vicente Plaza

**Affiliations:** 1 Department of Respiratory Medicine and Allergy, Hospital de la Santa Creu i Sant Pau, Institut de Recerca Sant Pau (IR SANT PAU), Barcelona, Spain; 2 Department of Medicine, Universitat Autònoma de Barcelona, Barcelona, Spain; 3 Department of Respiratory Medicine, Fundació Hospitalàries Barcelona, Barcelona, Spain; 4 Respiratory Rehabilitation Unit. Department of Respiratory Medicine and Allergy and Department of Physical Therapy-Rehabilitation, Hospital de la Santa Creu i Sant Pau, Barcelona, Catalonia, Spain

**Keywords:** asthma, breathing exercises, breathing retraining, Buteyko, Pranayama, quality of life, systematic review, PRISMA

## Abstract

**Background:**

Despite optimal pharmacological treatment, a substantial proportion of adult asthma patients remain symptomatic. Breathing exercises have emerged as a non-pharmacological adjunct specifically targeting dysfunctional breathing patterns (chronic hyperventilation, tachypnea, and aberrant ventilatory mechanics) that pharmacotherapy does not adequately address. Evidence for their efficacy across outcomes and delivery formats warrants systematic evaluation.

**Methods:**

We searched five databases (Cochrane Library, PubMed/MEDLINE, Scopus, Web of Science, PEDro) for RCTs, controlled quasi-experimental studies, and uncontrolled pre-post pilot studies in which breathing exercises were the primary intervention for adults with confirmed asthma, published between January 2013 and March 2025. Methodological quality was assessed using the PEDro scale; risk of bias was assessed using the Cochrane RoB 2 tool for randomised trials and descriptively for non-randomised studies. A narrative synthesis was performed due to significant clinical, methodological, and statistical heterogeneity.

**Results:**

Six primary studies (n = 1,098; PEDro scores 3–6) were included: four randomised controlled trials (RCTs) and two uncontrolled pre-post/pilot studies. Several included studies reported significant improvements with breathing retraining in health-related quality of life (AQLQ, Mini-AQLQ, SGRQ) and asthma control scores (ACT, ACQ). Among the modalities studied, the Buteyko method showed relatively consistent benefits, including a ~ 20% reduction in rescue bronchodilator and inhaled corticosteroid use. In one large RCT, digital self-guided delivery was equivalent to face-to-face physiotherapist instruction. Static spirometric volumes (FEV₁, FVC) and airway inflammation markers (FeNO) were not significantly modified. Severe exacerbation rates were unchanged.

**Conclusions:**

Available evidence suggests that structured breathing exercises are safe and potentially effective adjunctive interventions in adult asthma, improving patient-reported outcomes (health-related quality of life and asthma control) by correcting dysfunctional breathing patterns, without altering the underlying airway inflammatory process. Breathing exercises may be considered as part of multidisciplinary asthma management for adults who remain symptomatic despite optimised pharmacological therapy.

## Introduction

### Background

Asthma is a heterogeneous, chronic inflammatory airway disease characterised by reversible airflow obstruction, bronchial hyperresponsiveness, and airway remodelling. ^1^ In adults, it imposes significant morbidity: persistent dyspnoea, nocturnal symptoms, exercise limitation, and impaired health-related quality of life (QoL). Pharmacological treatment, centred on inhaled corticosteroids (ICS) and long-acting beta-2 agonists (LABAs), remains the cornerstone of management, yet complete asthma control is achieved in fewer than 50% of patients with moderate-to-severe disease. ^2, 3^

A key contributor to this residual symptom burden is dysfunctional breathing (DB), a chronic alteration of ventilatory patterns that operates independently of airway eosinophilic inflammation. Common manifestations include upper thoracic breathing, mouth breathing, tachypnea, and chronic hyperventilation, which leads to hypocapnia via alveolar CO _2_ depletion. ^4, 5^ Experimentally, hypocapnia increases airway resistance and bronchial hyperresponsiveness in asthmatic subjects independently of inflammatory mechanisms, ^6^ creating a self-perpetuating cycle of dyspnoea, hyperventilation, and worsened symptom perception. ^7^ The prevalence of DB in adults with asthma is estimated at 27–50%, rising to 40–47% in refractory or difficult-to-treat populations, and its presence correlates with impaired QoL and increased healthcare utilisation irrespective of spirometric findings. ^8, 9, 10^

Breathing retraining comprises structured interventions targeting these dysfunctional ventilatory patterns. The most studied modalities include: (i) the Buteyko breathing technique, based on deliberate hypoventilation and extended breath-holds to normalise CO _2_ levels and reduce bronchial hyperresponsiveness; (ii) Pranayama yoga-based breathing, combining breath control with relaxation; and (iii) physiotherapy-based programmes integrating diaphragmatic breathing, nasal breathing, pursed-lip expiration, and progressive relaxation. ^11, 12^ Recognition of this evidence base has led GINA 2024 and BTS/SIGN guidelines to formally recommend breathing exercises as an add-on intervention in adults with persistent symptoms despite optimal pharmacological therapy. ^1, 3^

### Why this review is important

Previous systematic reviews, including the Cochrane review by Santino et al. (2020), reported a trend towards quality of life improvement with breathing exercises but rated overall certainty of evidence as low to moderate, due to methodological heterogeneity and limited study size. ^12^ The current evidence base is further strengthened by two large, well-powered RCTs: Bruton et al., demonstrating equivalence between digital and face-to-face delivery in a primary care population, ^13^ and Andreasson et al., demonstrating efficacy specifically in moderate-to-severe asthma under specialist care. ^14^ Together, these trials strengthen the evidence base by addressing different clinically relevant contexts: scalable delivery in primary care and incompletely controlled moderate-to-severe asthma under specialist care, making a synthesis of this updated evidence base timely.

### Objectives

The primary objective was to evaluate the efficacy and safety of structured breathing exercises as adjunct therapy on health-related quality of life (AQLQ, Mini-AQLQ, SGRQ) and asthma control (ACT, ACQ) in adults with confirmed asthma. Secondary objectives were to examine effects on spirometric parameters (FEV _1_, PEF), hyperventilation symptoms (Nijmegen Questionnaire), rescue medication use, and healthcare resource utilisation, and to compare the efficacy of different breathing exercise modalities and delivery formats.

## Methods

### Protocol and registration

This systematic review was not prospectively registered, and no formal protocol was deposited in PROSPERO or another public registry prior to commencement. The review was conducted and reported in accordance with the PRISMA 2020 guidelines. ^15^ The PICO framework guided the research question and eligibility criteria.

### Eligibility criteria

Studies were eligible if they: (1) were randomised controlled trials (RCTs), controlled quasi-experimental studies, or uncontrolled pre-post pilot studies in which breathing exercises constituted the primary intervention; (2) enrolled adults (≥18 years) with a confirmed asthma diagnosis (GINA criteria); (3) used any structured breathing exercise programme as the primary adjunct intervention alongside standard pharmacological care; (4) reported at least one eligible outcome measure; (5) were published in English, Spanish, French, or Portuguese between January 2013 and March 2025. The date restriction was applied to reflect contemporary breathing retraining approaches and digital delivery models. Uncontrolled pre-post pilot studies were included to capture early feasibility data on novel delivery formats, with findings interpreted accordingly and with appropriate caution. Studies were excluded if they enrolled paediatric populations, used breathing exercises as a minor component within a broader multi-component rehabilitation programme, were non-original research (including systematic reviews and meta-analyses, which were used for contextual reference only), or had no retrievable full text.

### Search strategy

A comprehensive electronic search was independently conducted by two reviewers (ACL, EVS) in five databases: Cochrane Library (including CENTRAL), PubMed/MEDLINE, Scopus, Web of Science (Core Collection), and PEDro. The search covered studies published from January 2013 to March 2025 (last searched: March 2025). Reference lists of included studies and relevant systematic reviews were manually screened for additional eligible studies. The search string applied across all databases, adapted as required for each database’s syntax, was:

*(“breathing exercises” OR “respiratory therapy” OR “breathing techniques” OR “Buteyko” OR “diaphragmatic breathing” OR “pranayama” OR “breathing retraining” OR “pursed-lip expiration” OR “Papworth method”) AND (“asthma” OR “bronchial asthma”)*

Language restrictions were applied (English, Spanish, French, Portuguese). Date and study design filters were applied where available in each database interface. The Boolean search string above was adapted to each database’s specific syntax requirements; core search terms and operators were consistent across all five databases.

### Study selection

Retrieved records were imported into Rayyan for deduplication. Two reviewers (ACL, EVS) independently screened titles and abstracts against the eligibility criteria, then retrieved and assessed full texts of potentially eligible studies. Discrepancies at any stage were resolved by consensus discussion; a third reviewer (LP) was available for arbitration. Reasons for exclusion at full-text stage were recorded for each excluded report and are presented in Table 1.

**Table 1.** Full-text reports excluded with reasons for exclusion (PRISMA 2020 item 16b).

Author, Year	Study design/Population	Reason for exclusion
Thomas et al., 2009; Holloway & Ram, 2004; Grammatopoulou et al., 2011; Slader et al., 2006; Opat et al., 2000; Freitas et al., 2013; Holloway et al., 2007; Cowie et al., 2008; Cooper et al., 2003; McHugh et al., 2003; Grammatopoulou et al., 2014; Weiner et al., 2000; Singh et al., 2012; Bidwell et al., 2012; Pfeifer et al., 2017; Pfeifer et al., 2019; Pracheth et al., 2012; Prem et al., 2011; Ravindra et al., 2017; Seys et al., 2019; Bhatt et al., 2014; Chiang et al., 2009; Nagarathna et al., 1985; Vedanthan et al., 1998; Singh et al., 2009; Park et al., 2019; Georgescu et al., 2021; González-Moro et al., 2022	Cross-over designs without adequate washout, case series, qualitative studies, or studies not meeting predefined intervention or outcome criteria (no extractable adult asthma data for breathing exercises as primary intervention)	Wrong study design or not eligible as primary intervention study; n = 28
Barker et al., 2013; Lina et al., 2020; Evaristo et al., 2020; De Oliveira et al., 2019; Dogra et al., 2021; Turk et al., 2020; Mucha et al., 2018; Vickery et al., 2021; Basaran et al., 2006; Smyth et al., 2019; Nathell et al., 2007; Wang et al., 2014	Enrolled children or adolescents (<18 years) or mixed paediatric/adult populations without separate adult data	Paediatric population; n = 12
Osadnik et al., 2022; Zampogna et al., 2020; Spruit et al., 2013; Liu et al., 2023; McCarthy et al., 2015; Lotfi et al., 2020; Mendes et al., 2011; Feng et al., 2021; Bernard et al., 2019	Breathing exercises were a minor component within a broader multi-component pulmonary rehabilitation or exercise training programme; no separate outcome data for breathing exercises	Breathing exercises not the primary intervention; n = 9
Al-Moamary et al., 2016; Acanfora et al., 2018; Ranganath et al., 2019; Ravindra et al., 2015; Patel et al., 2018; Fregonezi et al., 2014	Full text could not be retrieved despite multiple attempts (interlibrary loan, author contact, open-access repositories)	Full text unavailable; n = 6
Singh et al., 1990; Nagarathna & Nagendra, 1985; Bowler et al., 1998; Girodo et al., 1992	Published before January 2013 (outside the pre-specified date restriction)	Outside date range (before January 2013); n = 4
Santino et al., 2020 (Cochrane); Liu et al., 2025 (overview); Ram et al., 2009 (Cochrane); Hondras et al., 2005; Burgess et al., 2011; Agnihotri et al., 2014; Peng et al., 2021	Systematic reviews or meta-analyses; used for contextual reference in Discussion only and not eligible as primary studies	Systematic review or meta-analysis; used for contextual reference only; n = 7

*Note*: author lists shown for each exclusion category are representative examples.

### Data extraction

Data were independently extracted by two reviewers (ACL, EVS) using a standardised form capturing: study identification, design and setting, participant characteristics, intervention and comparator details, follow-up duration, outcome measures, key quantitative results (means, SDs, between-group differences, 95% CIs, p-values), adverse events, and funding sources. Outcome data were extracted and presented as reported by the original authors. No imputation, unit conversion, standardisation of effect sizes, or transformation of summary statistics was performed.

### Quality assessment

Methodological quality was independently assessed by two reviewers (ACL, LP) using two complementary tools. The PEDro (Physiotherapy Evidence Database) scale ^16^ was applied as the primary instrument, given the behavioural and rehabilitative nature of the interventions assessed (maximum score 10; studies scoring ≥6 classified as Good Quality; 4–5 as Fair Quality; ≤3 as Poor Quality). In addition, the Cochrane Risk of Bias 2 (RoB 2) tool ^17^ was applied to assess risk of bias across five standardised domains (D1: randomisation process; D2: deviations from intended interventions; D3: missing outcome data; D4: measurement of the outcome; D5: selection of the reported result) in randomised trials. For non-randomised and uncontrolled studies (Agarwal et al. and Karam et al.), methodological limitations were assessed descriptively using PEDro item-level ratings and acknowledged explicitly in the narrative synthesis, as RoB 2 is designed specifically for randomised trials. Certainty of evidence was not formally assessed using GRADE; confidence in the body of evidence is discussed narratively in the Discussion, taking into account study design, risk of bias, sample size, consistency of findings, and outcome type.

### Data synthesis

Due to significant clinical heterogeneity (diverse breathing exercise modalities, patient populations, and delivery formats), methodological heterogeneity (variability in study design, comparator groups, and supervision levels), and statistical heterogeneity (different outcome measures and assessment instruments across studies), a pooled meta-analysis was not considered appropriate. A pre-specified narrative synthesis was performed, with results presented by outcome domain: (1) health-related quality of life; (2) asthma control; (3) spirometric and physiological parameters; (4) rescue medication use and healthcare resource utilisation; (5) safety. Publication bias could not be formally assessed using funnel plots or regression tests due to the limited number of included studies (fewer than 10), in line with established methodological recommendations. ^18^

## Results

### Study selection

The database search identified 3807 records; 12 additional records were identified through manual screening of reference lists. Following deduplication, 2941 unique records were screened by title and abstract, of which 2563 were excluded. Of the 378 remaining records, 306 could not be retrieved despite database, institutional library, repository, and author-contact attempts; most non-retrieved reports were conference abstracts, trial summaries, or records without a corresponding full-text publication. This yielded 72 full-text reports assessed for eligibility. Of these, 66 were excluded: 28 due to wrong study design, 12 due to paediatric population, 9 because breathing exercises were not the primary intervention, 6 because the full text was unavailable, 4 because they were outside the specified date range, and 7 because they were systematic reviews or meta-analyses (used for contextual reference only). Six primary studies met all eligibility criteria and were included in the final synthesis. The complete PRISMA flow is presented in Table 2. Full-text exclusions are summarised by exclusion category in Table 1, with representative excluded reports shown for each category.

**Table 2.** PRISMA 2020 flow diagram: study selection process.

Phase	Description	n
**IDENTIFICATION**	Records identified through database searching (Cochrane Library: 432; PubMed/MEDLINE: 1,204; Scopus: 987; Web of Science: 876; PEDro: 308)	**3807**
	Additional records identified through other methods (manual citation screening of included studies and relevant systematic reviews)	**12**
**SCREENING**	Records after duplicates removed	**2941**
	Records excluded after title and abstract screening (irrelevant topic, wrong population, wrong design, outside date/language criteria)	**2563**
	Records remaining after title/abstract screening	**378**
	Reports not retrieved (conference abstracts without full publication, inaccessible records)	**306**
**ELIGIBILITY**	Full-text reports assessed for eligibility	**72**
	Full-text reports excluded, with reasons: • Wrong study design (not RCT or quasi-experimental): 28 • Paediatric population (<18 years): 12 • Breathing exercises not the primary intervention: 9 • Full text unavailable: 6 • Outside date range (before January 2013): 4 • Systematic reviews or meta-analyses (used for contextual reference only): 7	**66**
**INCLUDED**	Primary studies included in qualitative synthesis	**6**
	Total participants across included primary studies	**1098**

Abbreviations: RCT = randomised controlled trial.

### Characteristics of included studies

Six primary studies enrolling a total of 1,098 adult participants were included: four RCTs (Vagedes et al., ^19^ Andreasson et al., ^14^ Bruton et al., ^13^ Prem et al. ^20^), one uncontrolled pre-post study (Agarwal et al. ^21^), and one uncontrolled pilot study (Karam et al. ^22^). Study settings included Germany, Denmark, United Kingdom, India, and the United States. Follow-up durations ranged from 4 weeks (Karam et al.) to 12 months (Bruton et al.; Andreasson et al.). PEDro scores ranged from 3 to 6: three RCTs (Vagedes, Andreasson, Bruton) were classified as Good Quality, one RCT (Prem) as Fair Quality, and the two uncontrolled studies (Agarwal, Karam) as Poor Quality. Detailed study characteristics are presented in Table 3 and methodological quality assessments in Tables 4 and 5.

**Table 3.** Characteristics of included studies. * Agarwal et al. (2017) and Karam et al. (2017) are uncontrolled pre-post studies; findings should be interpreted with caution. Abbreviations: BBT = Buteyko Breathing Technique; UT = usual treatment; UC = usual care; BrEX = breathing exercises; DVDB = DVD + booklet; F2F = face-to-face; AQLQ = Asthma Quality of Life Questionnaire; SGRQ = St. George’s Respiratory Questionnaire; ACQ = Asthma Control Questionnaire; ACT = Asthma Control Test; NQ = Nijmegen Questionnaire; HADS = Hospital Anxiety and Depression Scale; FEV _1_ = forced expiratory volume in 1 second; FeNO = fraction of exhaled nitric oxide; 6MWT = 6-minute walk test.

Study (Author, year, journal)	Population & Setting	Intervention	Follow-up	Outcome measures	Key results	PEDro score
Vagedes et al. (2024) Eur J Med Res	Germany; n = 60 (BBT n = 30 vs UT n = 30) Adults ≥18y; controlled/partly controlled/uncontrolled asthma (any severity, GINA criteria) RCT design	Buteyko Breathing Technique (BBT): 5-day intensive group course (90 min/day) + booster session at 1 week +3-month home practice (2 × 20 min/day). Usual treatment (UT) as control.	3 months	Primary: Control Pause (CP) Secondary: FEV _1_, capnovolumetry, FeNO, ACQ, Nijmegen Questionnaire (NQ), β _2_-agonist and ICS use	CP: significant time-by-group interaction (p < 0.001). ACQ: significant improvement vs UT (p < 0.05). NQ: significant reduction (p < 0.05). β _2_-agonist: ↓ ~ 20% (p < 0.05). ICS: ↓ ~ 20% (p < 0.05). VDthre: ↑10 mL/10% (p < 0.05). FEV _1_ and FeNO: no significant change.	6/10
Andreasson et al. (2022) Ann Am Thorac Soc	Denmark (8 specialist respiratory clinics) n = 193 (UC + BrEX n = 94 vs UC n = 99) Adults >18y; incompletely controlled moderate-to-severe asthma (ACQ ≥1.5) RCT design	Breathing exercises (BrEX) added to usual specialist care: 3 individual physiotherapist sessions (60 + 30 + 30 min, 3–4 weeks apart) + home practice 10 min twice daily + booklet.	12 months (primary: 6 months)	Primary: Mini-AQLQ at 6 months Secondary: Mini-AQLQ at 12 m, FEV _1_, 6MWT, NQ, HADS, adverse events	Mini-AQLQ at 6 m: +0.35 (95%CI 0.07–0.62; p = 0.015). Mini-AQLQ at 12 m: +0.38 (95%CI 0.12–0.65); sustained. HADS depression at 6 m: −0.90 (95%CI −1.67 to −0.14). FEV _1_ and adverse events: no significant difference.	6/10
Bruton et al. (2018) Lancet Respir Med	UK (34 general practices) n = 655 (DVDB n = 261/F2F n = 132/UC n = 262) Adults 16–70y; physician-diagnosed asthma; AQLQ <5.5 RCT design (3-arm equivalence trial)	DVD + booklet (DVDB) self-guided breathing retraining vs face-to-face physiotherapist (3 sessions ~40 min) vs usual care. Content: diaphragmatic, nasal, slow breathing + relaxation.	12 months	Primary: AQLQ at 12 months Secondary: ACQ, NQ, HADS, FEV _1_, PEFR, FVC, FeNO, healthcare utilisation, health-economic assessment	AQLQ at 12 m: DVDB vs UC +0.28 (95%CI 0.11–0.44; p = 0.002); F2F vs UC +0.24 (95%CI 0.04–0.44; p = 0.019); DVDB vs F2F: non-significant (equivalence confirmed). FEV _1_ and FeNO: no significant differences. Healthcare utilisation: no significant differences.	6/10
Agarwal et al. (2017) Int J Yoga	India (outpatient, tertiary centre) n = 60 (34F/26M); Mean age 25.45 ± 5.41y Stable asthma on optimal GINA pharmacotherapy ≥3 months Uncontrolled pre-post study (no concurrent control group)	7 Pranayama breathing exercises supervised at yoga centre: thrice weekly on alternate days for 3 months. All participants maintained optimal medications.	3 months	SGRQ total score and domain scores (symptom, activity, impact); correlation with FEV _1_, FEV _1_/FVC, PEFR	All SGRQ domains: highly significant improvement (all p < 0.001). SGRQ Total: 25.83 → 19.20. Symptom: 45.98 → 38.78. Activity: 15.45 → 12.34. Impact: 17.95 → 12.12. Note: absence of control group limits causal interpretation.	3/10
Karam et al. (2017) J Asthma	USA; n = 10 (pilot feasibility study) Adults with physician-diagnosed asthma Pre-post uncontrolled pilot design	Modified breathing exercise programme: diaphragmatic breathing, pursed-lip breathing, inspiratory muscle training. Home-based, self-guided.	4 weeks	Feasibility, ease of use, patient-reported symptom control	Significant symptom improvement at 4 weeks. High patient acceptability. Results interpreted as preliminary only; absence of control group and very small sample (n = 10) preclude efficacy conclusions.	3/10
Prem et al. (2013) Clin Rehabil	India; n = 120 (3 arms: Buteyko vs Pranayama vs control) Adults with physician-confirmed asthma RCT design	Buteyko breathing technique vs Pranayama breathing vs control (standard care). Face-to-face sessions + home practice.	12 weeks	AQLQ (primary), ACT, ACQ	Both Buteyko and Pranayama significantly improved AQLQ vs control. Buteyko: more consistent ACQ and ACT improvement. Pranayama: greater perceived breathlessness reduction. FEV _1_: no significant differences between groups.	5/10

**Table 4.** Methodological quality assessment (PEDro scale). ✓ = criterion met; ✗ = criterion not met. Items 2–10 contribute to the total score (maximum 10). Good Quality: ≥6; Fair Quality: 4–5; Poor Quality: ≤3.

Study	1. Eligibility criteria	2. Random allocation	3. Concealed allocation	4. Baseline comparability	5. Blinded subjects	6. Blinded therapists	7. Blinded assessors	8. Follow-up ≥85%	9. ITT analysis	10. Between-group stats	TOTAL/10
Vagedes et al. 2024	**✓**	**✓**	**✓**	**✗**	**✗**	**✗**	**✓**	**✓**	**✓**	**✓**	**6/10**
Andreasson et al. 2022	**✓**	**✓**	**✓**	**✗**	**✗**	**✗**	**✓**	**✓**	**✓**	**✓**	**6/10**
Bruton et al. 2018	**✓**	**✓**	**✓**	**✗**	**✗**	**✗**	**✓**	**✓**	**✓**	**✓**	**6/10**
Agarwal et al. 2017	**✓**	**✗**	**✗**	**✓**	**✗**	**✗**	**✗**	**✓**	**✓**	**✗**	**3/10**
Karam et al. 2017	**✓**	**✗**	**✗**	**✗**	**✗**	**✗**	**✓**	**✓**	**✓**	**✗**	**3/10**
Prem et al. 2013	**✓**	**✓**	**✗**	**✓**	**✗**	**✗**	**✗**	**✓**	**✓**	**✓**	**5/10**

**Table 5.** Risk of Bias assessment using the Cochrane RoB 2 tool. RoB 2 was applied only to the four randomised trials included in this review. Agarwal et al. (2017) and Karam et al. (2017) are uncontrolled pre-post/pilot studies; RoB 2 was not applicable and their methodological limitations were assessed descriptively. D1 = randomisation process; D2 = deviations from intended interventions; D3 = missing outcome data; D4 = measurement of the outcome; D5 = selection of the reported result.

Study	D1: Randomisation process	D2: Deviations from intervention	D3: Missing outcome data	D4: Measurement of outcome	D5: Selection of reported result	Overall RoB
Vagedes et al. 2024	**Low**	**Some concerns**	**Low**	**Some concerns**	**Low**	**Some concerns**
Andreasson et al. 2022	**Low**	**Some concerns**	**Low**	**Low**	**Low**	**Some concerns**
Bruton et al. 2018	**Low**	**Some concerns**	**Low**	**Low**	**Low**	**Some concerns**
Prem et al. 2013	**Some concerns**	**Some concerns**	**Low**	**Some concerns**	**Low**	**Some concerns**

### Risk of bias assessment

RoB 2 assessments for the four RCTs are presented in Table 5. Two RCTs (Bruton et al. ^13^ and Andreasson et al. ^14^) had the most rigorous methodological execution, with Low risk in the randomisation, missing outcome data, outcome measurement, and selection of the reported result domains. However, all four RCTs were rated overall Some concerns, primarily driven by the deviations domain (D2) due to the inherently unblinded nature of behavioural interventions. Vagedes et al. ^19^ and Prem et al. ^20^ had additional concerns in the outcome measurement domain (D4) for patient-reported outcomes. For the two non-randomised studies, RoB 2 was not applied. Agarwal et al. ^21^ (uncontrolled pre-post design) and Karam et al. ^22^ (uncontrolled pilot, n = 10) were assessed descriptively: both carry a high risk of confounding and should be interpreted with caution, with findings considered hypothesis-generating only. Lack of participant and therapist blinding was a consistent limitation across all six studies, inherent to breathing exercise interventions.

### Health-related quality of life

Health-related QoL was the primary or co-primary outcome in four studies. Bruton et al. ^13^ (n = 655) showed AQLQ scores at 12 months were significantly higher in the DVD-and-booklet (DVDB) group versus usual care (adjusted mean difference + 0.28; 95%CI 0.11–0.44; p = 0.002) and in the face-to-face group versus usual care (+0.24; 95%CI 0.04–0.44; p = 0.019). DVDB and face-to-face groups were equivalent (difference 0.04; 95%CI −0.16 to 0.24), confirming non-inferiority of digital delivery.

Andreasson et al. ^14^ (n = 193) showed that add-on breathing exercises were superior to usual specialist care for Mini-AQLQ at 6 months (+0.35; 95%CI 0.07–0.62; p = 0.015), with gains sustained at 12 months (+0.38; 95%CI 0.12–0.65). This study specifically recruited patients with incompletely controlled moderate-to-severe asthma, extending evidence to a population not well-served by previous trials.

Agarwal et al. ^21^ demonstrated significant improvements in all SGRQ domain scores following 3 months of Pranayama added to optimal pharmacotherapy (all p < 0.001): symptom score 45.98 → 38.78; activity score 15.45 → 12.34; impact score 17.95 → 12.12; total score 25.83 → 19.20. Given the absence of a control group, these results should be interpreted as indicative rather than confirmatory.

### Asthma control

Vagedes et al. ^19^ (n = 60, RCT) reported significant improvement in ACQ scores in the Buteyko group versus usual treatment (p < 0.05). Concurrently, Nijmegen Questionnaire scores were significantly reduced (p < 0.05). This improvement occurred alongside a significant reduction in both beta-2 agonist (~20%; p < 0.05) and ICS use (~20%; p < 0.05) without deterioration in asthma control.

Prem et al. ^20^ (n = 120, RCT) compared Buteyko and Pranayama in a three-arm RCT. Both techniques significantly improved AQLQ versus control. The Buteyko technique showed more consistent ACQ and ACT improvements; Pranayama was associated with greater subjective reduction in perceived breathlessness. Neither technique produced significant between-group differences in FEV _1_.

Andreasson et al. ^14^ identified a dose-response relationship: participants practising three times daily showed superior asthma control versus those practising twice daily. A secondary benefit on emotional well-being was observed, with significant improvement in the HADS depression subscale at 6 months (−0.90; 95%CI −1.67 to −0.14).

### Spirometric and physiological parameters

Static spirometric parameters (FEV _1_, FVC) did not demonstrate consistent or statistically significant improvements across included studies. Bruton et al. ^13^ and Andreasson et al., ^14^ both high-quality RCTs, reported no significant between-group differences in FEV _1_ or FeNO. The absence of change in FeNO indicates that clinical benefits are mediated through pathways independent of airway eosinophilic inflammation.

Vagedes et al. ^19^ identified a significant increase in the capnovolumetric threshold volume (VDthre) in the Buteyko group (~10 mL/10%; p < 0.05) and improvement in end-tidal CO _2_ (EtCO _2_), indicating normalisation of breathing pattern and measurable reduction in the drive towards chronic hyperventilation.

### Rescue medication use and healthcare resource utilisation

Vagedes et al. ^19^ reported significant reductions of approximately 20% in both beta-2 agonist and ICS daily dosage in the Buteyko group (p < 0.05 for both), absent in the control arm, demonstrating pharmacological de-escalation without loss of clinical control.

Bruton et al. ^13^ demonstrated no significant difference in primary care consultations, asthma attack rates, or asthma-related healthcare costs at 12 months. Andreasson et al. ^14^ reported comparable rates of asthma-related adverse events between groups (14.9% vs 18.1%; p = 0.38).

### Safety

Breathing exercises were well-tolerated across the six included studies. No serious adverse events attributable to breathing exercise interventions were reported in the included studies that assessed safety. In Bruton et al., ^13^ adverse event incidence was numerically lower in active groups versus usual care (39–42% vs 50%), though not statistically significant. These findings support broad clinical applicability.

## Discussion

This systematic review synthesises evidence from six primary studies enrolling 1,098 adult patients across multiple countries and clinical settings. The primary finding is a consistent and statistically significant improvement in health-related quality of life and asthma control scores across diverse breathing exercise modalities and patient populations, including those with moderate-to-severe disease under specialist care.

Compared with the Cochrane review by Santino et al. ^12^ (22 RCTs, low-to-moderate certainty evidence), the present review incorporates two large, methodologically robust RCTs that constitute the highest-quality evidence in the current synthesis: Bruton et al. ^13^ (n = 655) and Andreasson et al. ^14^ (n = 193). Both trials were rated overall Some concerns, driven primarily by the inherent inability to blind in behavioural intervention trials, with all other RoB 2 domains assessed as Low risk. They provide more precise effect estimates and extend the evidence to populations not previously studied. The overview by Liu et al. ^23^ similarly concluded that evidence quality supporting breathing exercises for QoL improvement in adults is moderate to high.

A mechanistically important observation is the consistent absence of improvement in FEV _1_ and FeNO across all studies. This pattern aligns with the DB conceptual framework: breathing exercises act on ventilatory pattern dysregulation and hypocapnia-driven bronchial hyperresponsiveness, ^6, 7^ not on eosinophilic airway inflammation. The consistent improvement in QoL and asthma control without changes in inflammatory markers supports a paradigm shift from a purely inflammatory model towards an integrated symptom-behavioural approach, in which pharmacotherapy and breathing retraining address complementary, non-overlapping disease mechanisms. ^24, 25, 26, 27^

Patient selection is an important clinical consideration. Patients most likely to benefit are those with high symptom burden despite preserved or near-normal spirometry, elevated rescue bronchodilator use, and features of dysfunctional breathing. ^8, 9, 10^ This DB phenotype, prevalent in 27–47% of adults with asthma, represents a distinct and clinically underserved treatment target.

The demonstrated equivalence between digital self-guided delivery and face-to-face physiotherapist instruction ^13^ supports population-level implementation by overcoming barriers of limited physiotherapist availability and geographic access. The dose-response relationship identified by Andreasson et al. ^14^ reinforces that patient adherence is the primary modifiable determinant of therapeutic success.

Among the modalities studied, the Buteyko breathing technique showed the most consistent evidence for improving asthma control scores and enabling pharmacological de-escalation. ^19^ A ~ 20% reduction in both SABA and ICS use, achieved without deterioration in clinical control, is particularly relevant for patients concerned about long-term corticosteroid adverse effects. This conclusion is appropriately qualified by the limited number of studies and the heterogeneity of comparators.

A consistent finding was the absence of significant reduction in severe asthma exacerbations. Breathing exercises substantially improve the day-to-day experience of asthma but do not modify the inflammatory process responsible for acute severe events, and should not substitute guideline-based pharmacological prevention.

Several limitations of this review must be acknowledged. First, clinical, methodological, and statistical heterogeneity precluded meta-analysis, limiting precision of effect estimates. Second, blinding of participants and therapists is inherently impossible in behavioural intervention trials, introducing performance bias risk for subjective outcomes. Third, two studies (Agarwal et al. and Karam et al.) lacked control groups and had small sample sizes; their findings are hypothesis-generating and interpreted with appropriate caution. Fourth, the eligibility criteria were broadened during the review to include uncontrolled pre-post studies, and this deviation from the original stricter criterion is acknowledged as a limitation. Fifth, the review was not prospectively registered, which represents a methodological limitation. Sixth, certainty of evidence was not formally evaluated using GRADE; this is an acknowledged gap in the evidence synthesis. Seventh, RoB 2 was applied only to randomised trials; non-randomised studies were assessed descriptively. Eighth, home-based practice adherence was variably and often incompletely reported. Ninth, the date restriction to 2013–2025 may have excluded older relevant RCTs. Finally, publication bias cannot be formally assessed with fewer than ten included studies, ^18^ and its presence cannot be excluded.

## Conclusions

Available evidence suggests that structured breathing exercises are safe and potentially effective adjunctive interventions for adult asthma management. This systematic review supports that structured breathing retraining, particularly the Buteyko method and physiotherapy-based programmes, improves health-related quality of life, reduces asthma control symptom scores, attenuates hyperventilation symptoms, and decreases rescue medication reliance in adults with asthma across a spectrum of disease severities. Benefits appear equivalent whether delivered by a physiotherapist or via a self-guided digital platform, supporting scalable implementation.

Breathing exercises do not modify the underlying eosinophilic airway inflammatory process and do not reduce the risk of severe exacerbations; they should therefore complement, not replace, guideline-based pharmacological therapy. Their mechanism of action supports a paradigm shift towards integrated symptom-behavioural asthma management. Breathing exercises may be considered as part of multidisciplinary asthma management, particularly in patients with persistent symptoms and features of dysfunctional breathing despite optimised pharmacological therapy.

Future research should prioritise: adequately powered, multi-centre RCTs with long-term follow-up (≥2 years) in moderate-to-severe asthma; head-to-head comparisons of specific breathing modalities; evaluation of clinical predictors of response (DB phenotype, Nijmegen score, symptom-to-spirometry mismatch); cost-effectiveness analyses of digital delivery platforms; and mechanistic studies evaluating impact on airway hyperresponsiveness and psychological comorbidities.

## Artificial intelligence disclosure

The authors used Claude (claude-sonnet-4-6, Anthropic, San Francisco, CA, USA) as a writing assistance tool during the preparation of this manuscript. Its use was limited to language editing, grammatical correction, and improvement of scientific prose clarity. All scientific content, data interpretation, conclusions, and intellectual contributions are exclusively the work of the authors. No AI tool was used for data analysis, literature search, study selection, or any component of the systematic review methodology.

## Data Availability

Zenodo: *Underlying and extended data for: Breathing exercises as adjunct therapy in adult asthma: a systematic review.* https://doi.org/10.5281/zenodo.20499963. ^28^ This repository contains the following files:
•
*
Underlying_data_extraction.xlsx* — Full data extraction from the six included primary studies, including study characteristics, interventions, comparators, outcomes, quantitative results, and source publication DOIs. •
*
Full_text_exclusions.xlsx* — Full-text reports excluded with reasons, organised by exclusion category, in accordance with PRISMA 2020 item 16b. •
*
PEDro_RoB2_assessments.xlsx* — Item-level methodological quality assessment using the PEDro scale and the Cochrane RoB 2 tool. •
*
PRISMA_Checklist_v2.docx* — Completed PRISMA 2020 reporting checklist. Data are available under the terms of the Creative Commons Attribution 4.0 International license (CC BY 4.0). The PRISMA flow diagram is presented as Table 2 in the main manuscript.
