# Breathing Exercises as Adjunct Therapy in Adult Asthma: A Systematic Review

**DOI:** 10.12688/f1000research.180230.2

**Published:** 2026-10-31

**Authors:** Astrid Crespo-Lessmann, Eduardo Vélez-Segovia, Luis Paz, Vicente Plaza

**Affiliations:** 1 Department of Respiratory Medicine and Allergy, Hospital de la Santa Creu i Sant Pau, Institut de Recerca Sant Pau (IR SANT PAU), Barcelona, Spain; 2 Department of Medicine, Universitat Autònoma de Barcelona, Barcelona, Spain; 3 Department of Respiratory Medicine, Fundació Hospitalàries Barcelona, Barcelona, Spain; 4 Respiratory Rehabilitation Unit. Department of Respiratory Medicine and Allergy and Department of Physical Therapy-Rehabilitation, Hospital de la Santa Creu i Sant Pau, Barcelona, Catalonia, Spain

**Keywords:** asthma, breathing exercises, breathing retraining, Buteyko, Pranayama, quality of life, systematic review, PRISMA

## Abstract

**Background:**

Persistent symptoms and impaired quality of life can remain despite pharmacological treatment of asthma. Breathing retraining may address an additional component of this burden. We examined whether reported benefits are consistent across outcomes, interventions and settings, while distinguishing comparative evidence from uncontrolled observations.

**Methods:**

The original review reported searches of Cochrane Library, PubMed/MEDLINE, Scopus, Web of Science and PEDro for studies published from January 2013 to March 2025. Randomised trials and uncontrolled pre-post studies were included. A supplementary targeted search and full-text review identified four additional eligible trials (three randomised and one quasi-randomised) that had not been incorporated in the original selection; these were checked against the review’s eligibility criteria and added. A further updated bibliographic search conducted in October 2026, restricted to the original January 2013–March 2025 window, identified five more eligible randomised trials that had likewise not been incorporated; these were also checked against the same eligibility criteria and added. In this revision, reported results were checked against source publications and synthesised by outcome and design. PEDro ratings were corrected; limitations of the original RoB 2 assessments and selection records are disclosed. Meta-analysis and formal GRADE assessment were not performed.

**Results:**

Fifteen studies enrolled 1,786 participants with asthma: twelve randomised trials, one quasi-randomised trial, and two uncontrolled studies. Quality-of-life benefits relative to usual care or a non-active control were supported by Bruton, Andreasson, Prem, Rondinel, Sodhi and von Bonin; against active comparators, one additional trial favoured breathing exercises over progressive relaxation, three found no significant difference against aerobic training, a sham control or combined aerobic exercise, and two found no statistically significant between-group difference between two active breathing-based techniques. The uncontrolled Karam study did not show significant Mini-AQLQ improvement. Asthma-control findings were mixed, with no significant between-group ACQ benefit in Bruton or Andreasson, a significant benefit of incentive spirometry with expiratory positive-airway pressure over usual care in Rondinel, while one additional trial favoured a breathing technique over relaxation on the Asthma Control Test and two further trials against active comparators found no significant between-group difference in asthma-control scores. One trial demonstrated equivalence of DVD-and-booklet and face-to-face delivery of the same programme. Lung-function and FeNO benefits were not established, and one trial found a greater reduction in rescue-medication use with aerobic training than with breathing exercises. Safety evidence remained limited. The completeness of the original study selection could not be fully verified, although the eligibility of nine previously unresolved or newly identified candidate reports has now been checked and resolved across two targeted audits, including an updated database search.

**Conclusions:**

Structured breathing retraining may improve health-related quality of life as an adjunct to asthma pharmacotherapy relative to usual care, although comparative evidence against active comparators is more mixed. Evidence for improved patient-reported asthma control is similarly inconsistent. Heterogeneity, uncontrolled designs, and incomplete original search documentation limit confidence and generalisability; RoB 2 classified nine of the twelve randomised trials at high overall risk of bias and three as having some concerns, limiting confidence in the apparent benefits. The evidence does not establish superiority of one technique, a medication step-down strategy, or prevention of exacerbations.

## Introduction

### Background

Asthma is a heterogeneous inflammatory airway disease associated with variable respiratory symptoms and airflow limitation. Pharmacological treatment remains central to management, but symptoms and impaired health-related quality of life (QoL) may persist despite treatment. ^1^ ^–^ ^3^ Asthma also carries a substantial population burden. ^11^ Adjunctive interventions should therefore be evaluated against patient-important outcomes and their limitations made explicit.

Dysfunctional breathing describes alterations in breathing pattern that may contribute to breathlessness and symptom burden in people with asthma. It encompasses several patterns; hyperventilation with hypocapnia is one possible presentation and is not present in every patient. ^4^ ^–^ ^8^ Observational studies link breathing-pattern disturbance with impaired health status in difficult-to-treat asthma. ^9^ ^,^ ^10^ This provides a rationale for evaluating breathing retraining, but does not establish which patients respond or whether benefit is mediated by changes in carbon dioxide.

Structured programmes include Buteyko breathing, pranayama and physiotherapy-based retraining using components such as diaphragmatic, nasal or slower breathing and relaxation. ^12^ Their content, supervision and home-practice requirements vary. GINA 2024 describes breathing exercises as a possible supplement to pharmacotherapy for symptoms and quality of life. ^1^ Guideline-based drug treatment remains necessary.

### Why this review is important

Santino et al. (2020) reported possible benefits of breathing exercises, with outcome-specific certainty ranging from moderate to very low; their search ended on 4 April 2019. ^12^ The BREATHE trial reported by Bruton et al. ^13^ was already included in that review under the study identifier Thomas 2017. It should therefore not be presented as newly available evidence. Later trials, including Andreasson et al. ^14^ in specialist care and Vagedes et al. ^19^ evaluating Buteyko breathing, provide a reason to revisit the evidence. The clinically relevant question is whether improvements in quality of life are accompanied by better asthma control, physiological changes or reduced medication use, and how differences in setting and delivery affect interpretation.

### Objectives

The primary objective was to evaluate reported effects of structured breathing exercises added to pharmacological care on health-related QoL (AQLQ, Mini-AQLQ and SGRQ) and patient-reported asthma control (ACT and ACQ). Secondary objectives were to examine lung function, symptoms assessed using the Nijmegen Questionnaire, medication use, healthcare utilisation and safety, and to identify the limits of comparisons between techniques and delivery formats.

## Methods

### Protocol and registration

The review was not prospectively registered, and no public protocol was deposited before commencement. PRISMA 2020 was used to guide reporting. ^15^ The available records do not establish that all methodological decisions were prespecified. This revision explicitly reports the undocumented eligibility amendment and gaps in reproducibility.

### Eligibility criteria

The published review specified randomised controlled trials (RCTs), controlled quasi-experimental studies and uncontrolled pre-post studies of structured breathing exercises added to pharmacological care, with at least one eligible outcome. It specified adults aged ≥18 years with confirmed asthma and publications in English, Spanish, French or Portuguese from January 2013 to March 2025. Breathing exercises had to be a principal intervention; non-original research and multicomponent programmes without separable breathing-exercise data were excluded. The date restriction was intended to emphasise contemporary programmes, but excludes older relevant evidence. There were no controlled quasi-experimental studies among the six selected reports. The inclusion of uncontrolled studies represented a change from a stricter design criterion, but the timing, decision process and any rescreening cannot be verified from the retained documents. These studies are therefore presented separately as exploratory evidence. Bruton enrolled participants aged 16–70 years and did not provide separate adult-only results; it is retained as an explicit exception, not documented as prospectively planned, because 16–17-year-olds represented a small minority of a sample recruited through adult respiratory clinics and because GINA does not define a separate paediatric stratum above early adolescence, so excluding the trial on this basis alone was judged to discard clinically relevant, largely adult evidence rather than to correct a design flaw. Eligibility by publication date used a single rule: the study’s formal journal publication (print/issue) date, not its online-first availability. Prem was available online in July 2012 but formally published in print in February 2013, and is retained within the January 2013–March 2025 window on that basis; this print-date rule is applied consistently to every other included study. Asthma diagnostic definitions also differed across studies and were not uniformly based on GINA criteria. These discrepancies are disclosed rather than retrospectively described as prespecified eligibility rules.

### Search strategy

The original review reported searches by two reviewers (ACL and EVS) in Cochrane Library (including CENTRAL), PubMed/MEDLINE, Scopus, Web of Science Core Collection and PEDro, with reference-list screening. The reported search period was January 2013 to March 2025, and the last search was reported as March 2025. Exact search dates and database-specific executed strategies were not retained among the available documents. The following core search expression was reported:

(“breathing exercises” OR “respiratory therapy” OR “breathing techniques” OR “Buteyko” OR “diaphragmatic breathing” OR “pranayama” OR “breathing retraining” OR “pursed-lip expiration” OR “Papworth method”) AND (“asthma” OR “bronchial asthma”)

The reported restrictions were English, Spanish, French or Portuguese, the publication period January 2013–March 2025, and study-design filters where available. The precise filters and adaptations used in each database cannot be reconstructed from the core expression alone. Source checking performed for this revision in October 2026 comprised two steps. First, a targeted audit of the manuscript and selected reports already in hand was conducted, as described above; this was not a repeat of the original five-database search or an extension of its search end date. Second, an updated, dated and reproducible PubMed/MEDLINE search was run on 12 October 2026 using the reported core search expression, restricted to the original January 2013–March 2025 publication window. Full-text assessment of the candidate reports it retrieved confirmed five further eligible randomised trials not present in the original selection (Rondinel, ^32^ Sodhi, ^33^ von Bonin, ^34^ Chung ^35^ and Kim ^36^); other candidate reports identified by this updated search were excluded as outside the review’s declared eligibility criteria or not accessible in full text. This updated search also failed to retrieve Ritz,, ^26^ a study already confirmed eligible and included in this review, indicating that the original search strategy or its database-specific execution may not have had complete sensitivity even for an included study; this is disclosed as an open limitation rather than resolved. The updated search was restricted to PubMed/MEDLINE and to the original review’s declared date window; it does not repeat the search in Cochrane Library, Scopus, Web of Science or PEDro, and does not extend the evidence base beyond the originally declared period.

### Study selection

The original manuscript described Rayyan deduplication and independent title/abstract and full-text screening by ACL and EVS, with consensus and LP available for arbitration. However, the individual record-level decisions and a dated amendment log are unavailable. The supplied exclusion file contains aggregate categories and author-year examples rather than a uniquely identified report-level log. The original flow and its discrepancies are shown in Tables 1 and 2; its completeness remains unverified.

**Table 1.** Audit of the reported exclusion summary. Counts are historical and have not been validated against individual records. This is not a complete PRISMA item 16b exclusion log.

Reported category	Reported n	Audit finding
Wrong design or ineligible intervention study	28	Author-year examples are not unique full citations; several pre-2013 reports overlap with the date exclusion rule. Reasons cannot be verified individually.
Paediatric population	12	The identified Evaristo 2020 RCT enrolled adults aged 30–65. The reported paediatric reason was unsupported for that report; on reassessment against the review’s eligibility criteria the study was confirmed eligible and is now included ( Table 3).
Breathing not the principal intervention	9	Aggregate category only; no report-level decisions available.
Full text unavailable	6	Belongs to the not-retrieved stage, not among reports assessed in full text. Counts cannot be corrected from arithmetic alone.
Outside date range	4	Possible overlap with earlier categories; unique records unavailable.
Systematic review or meta-analysis	7	Liu 2025 was published in July, after the reported March search end date; it cannot be counted as an exclusion from that search without a documented later search.
Reported total	66	Retained historical total, not a validated unique report count.

**Table 2.** Historical reported selection flow and unresolved audit issues. Figures are retained for traceability; no corrected final flow is asserted.

Selection step	Reported n	Status
Database records	3,807	432 Cochrane; 1,204 PubMed; 987 Scopus; 876 Web of Science; 308 PEDro. Executed searches unavailable.
Other-source records	12	Manual reference screening reported; individual records unavailable.
Records after deduplication	2,941	Historical count; implied duplicate removals = 878, not independently confirmed.
Title/abstract exclusions	2,563	Historical count; decision log unavailable.
Reports sought	378	Historical count.
Reports not retrieved	306	Historical count; six additional unavailable reports were classified elsewhere.
Reports assessed	72	Historical count; inconsistent with unavailable-report category.
Reports excluded at full text	66	Historical count; includes six unavailable reports and unverified examples.
Studies originally included	6	Corrected enrolled/randomised asthma sample across these reports = 1,162. The final eligible study set from the original search and screening remains unresolved (see next row for this revision’s targeted eligibility audit).
Studies included in this revision	15	Four previously unresolved candidate reports (Evaristo, Ritz, Coulson, and Erdoğan Yüce and Taşcı) and five further candidate reports identified by an updated PubMed/MEDLINE search conducted for this revision (Rondinel, Sodhi, von Bonin, Chung and Kim) were checked against the review’s eligibility criteria using their full texts and confirmed eligible; corrected enrolled/randomised asthma sample across all fifteen included reports = 1,786. The updated search also failed to retrieve Ritz, an already-included study, indicating a possible sensitivity limitation of the original search strategy. This resolves the status of these nine reports but does not reconstruct the original search and screening or confirm that no other eligible report remains unidentified.

### Data extraction

The original review described independent extraction by ACL and EVS using a standardised form for study design, setting, participants, interventions, comparators, follow-up, outcomes, effect estimates, adverse events and funding. During revision, the extraction table was checked against accessible source publications, correcting participant counts and unsupported outcome statements. Between-group estimates are distinguished from within-group changes. Mean differences, confidence intervals and p-values are reproduced in their original scales; no pooled estimate, imputation or standardisation was calculated for this review. The present source check does not replace the missing original selection log.

### Quality assessment

The original manuscript reported independent PEDro and RoB 2 assessment by ACL and LP. On checking the supplied assessment file, the PEDro matrix omitted item 11 and several ratings differed from confirmed PEDro database records.16 Table 4 now reports all 11 items: item 1 concerns eligibility and is excluded from the 0–10 total; items 2–11 contribute to the score. Confirmed database ratings are used for Bruton, Andreasson and Prem; the Vagedes rating is a provisional, source-based editorial appraisal that remains unvalidated. Under the descriptive thresholds previously used in this review, studies scoring ≥6 were classified as good quality; these thresholds do not establish certainty of treatment effects. Uncontrolled studies are assessed narratively without a PEDro total. RoB 2 is outcome-specific and applies to randomised trials.17 Formal outcome-specific RoB 2 assessment, with signalling-question-level justification archived in the Zenodo data deposit,28 is now reported in Table 5 for the review’s primary patient-reported outcome across all twelve randomised trials. Lack of blinding alone does not determine bias due to deviations from intended interventions. GRADE was not formally applied, and no formal certainty category is assigned in this revision.

### Data synthesis

Studies were grouped by outcome domain and study design. Comparative RCT estimates were considered first; uncontrolled pre-post changes were then described separately. Tables show sample size, intervention, comparator, follow-up and methodological limitations. Clinical and methodological differences in populations, programmes, comparators, outcome instruments and assessment times motivated narrative synthesis rather than an overall pooled effect. Statistical heterogeneity was not quantified because no meta-analysis was performed; variation in instruments is not itself a statistical heterogeneity estimate. Findings were compared for direction, magnitude and precision, including nonsignificant results, rather than counting statistically significant studies as proof of consistency. No subgroup meta-analysis, meta-regression or sensitivity analysis was undertaken. A funnel-plot or regression-based assessment of publication bias was not performed; the small number of studies and absence of pooling limit those methods. ^18^ Missing studies and selective reporting remain possible.

## Results

### Study selection

The published flow reported 3,807 database records and 12 records from other sources, 2,941 records after deduplication, 2,563 exclusions at title/abstract stage, 378 reports sought, 306 not retrieved, 72 assessed and 66 excluded, leaving six studies. These are historical reported figures rather than independently verified counts, and a classification inconsistency remains: six reports described as having unavailable full text were also counted among reports assessed at full text. The exclusion examples had also listed Evaristo et al. (2020) under a paediatric category, although the identified trial enrolled adults, and ^29^ Ritz et al. (2014), ^26^ Coulson et al. (2022) ^30^ and Erdoğan Yüce and Taşcı (2020) ^31^ were absent from the selection without a documented exclusion reason. On assessment against the eligibility criteria stated above, all four reports were confirmed eligible (randomised or quasi-randomised trials of structured breathing exercises in adults with confirmed asthma, published within the review’s date range, reporting an eligible outcome) and are added to the originally selected six studies, for ten included studies in total. This targeted audit does not reconstruct the original screening log, correct the unresolved classification inconsistency for the six originally selected studies, or confirm that no further eligible report remains unidentified; it resolves only the specific eligibility questions raised for these four reports. The following synthesis therefore covers ten studies and should not be treated as an independently validated exhaustive evidence base.

A further updated PubMed/MEDLINE search conducted for this revision (Methods) identified five additional eligible randomised trials that had not been part of either the original selection or the first targeted audit: Rondinel et al. (2014), ^32^ Sodhi et al. (2014), ^33^ von Bonin et al. (2018), ^34^ Chung et al. (2021) ^35^ and Kim et al. (2024). ^36^ These were assessed against the same eligibility criteria using their full texts and are added to the ten studies above, for fifteen included studies in total. Sodhi enrolled participants from 17 years and von Bonin enrolled participants from 12 years; both are retained as a declared limitation, for the same reason given above for Bruton, rather than excluded, because both trials recruited predominantly adult samples and did not report separable paediatric-only results. This second update does not reconstruct the original screening log, confirm the complete sensitivity of the original five-database search, or establish that no further eligible report remains unidentified; it resolves only the eligibility of these five reports. The following synthesis therefore covers fifteen studies and should not be treated as an independently validated exhaustive evidence base.

### Characteristics of included studies

The fifteen included studies enrolled 1,786 participants with asthma: twelve RCTs (Bruton, ^13^ Andreasson, ^14^ Vagedes ^19^ and Prem, ^21^ Evaristo, ^29^ Ritz, ^26^ and Erdoğan Yüce and Taşcı, ^31^ Rondinel, ^32^ Sodhi, ^33^ von Bonin, ^34^ Chung ^35^ and Kim ^36^), one quasi-randomised trial (Coulson ^30^), and two uncontrolled pre-post studies (Agarwal ^20^ and Karam ^22^). The total is the sum of enrolled/randomised asthma samples, not the number completing every outcome assessment. Karam enrolled 74 participants, of whom 68 completed follow-up; it was not a 10-person pilot. The 30 healthy participants in Vagedes are excluded from this total. Bruton allowed ages 16–70, so the aggregate is not an exclusively verified adult sample; Coulson restricted enrolment to adults aged 65 years or older. Unlike the other included trials, Evaristo compared breathing exercises with an active aerobic-training comparator rather than usual care; Erdoğan Yüce and Taşcı and Coulson compared breathing exercises with an active relaxation technique or a sham incentive-spirometer control; and Ritz compared two active breathing techniques directly, without a non-breathing control arm. Among the five further included trials, Rondinel and Sodhi compared a breathing-based intervention with usual care; von Bonin used a crossover design against a non-active waiting-list control; and Chung and Kim compared two active interventions directly (conventional breathing exercises against inspiratory muscle training, and aerobic exercise combined with Buteyko breathing against aerobic exercise combined with muscle stretching, respectively), without a non-breathing or usual-care arm. Follow-up ranged from one to twelve months, and populations, diagnostic definitions, comparators and supervision differed. Table 3 summarises characteristics. Confirmed PEDro scores are 7/10 for Bruton, Andreasson and Prem, 8/10 for Evaristo and Erdoğan Yüce and Taşcı, and 6/10 for Ritz and Coulson; the provisional Vagedes score is 6/10; among the five further included trials, confirmed PEDro scores are 8/10 for Kim, 7/10 for Chung, 5/10 for Rondinel and von Bonin, and 4/10 for Sodhi ( Table 4).

**Table 3.** Characteristics of the fifteen included studies. Enrolled/randomised asthma samples total 1,786; completers are not substituted for enrolled samples. Healthy controls in Vagedes are excluded. QoL = quality of life; AQLQ = Asthma Quality of Life Questionnaire; ACQ = Asthma Control Questionnaire; ACT = Asthma Control Test; SGRQ = St George’s Respiratory Questionnaire; PImax = maximal inspiratory pressure.

Study and design	Asthma sample	Population and setting	Programme and comparator	Follow-up	Principal verified finding
Bruton 2018 ^13^ Three-arm RCT	655 DVDB 261; face-to-face 132; usual care 262	UK primary care; ages 16–70; physician-diagnosed asthma; AQLQ <5.5	DVD/booklet or three physiotherapist sessions; usual care comparator	12 months	AQLQ favoured both programmes. Delivery arms equivalent within prespecified margin. ACQ, FEV _1_ and FeNO differences not significant.
Andreasson 2022 ^14^ RCT	193 BrEX 94; usual care 99	Eight Danish specialist clinics; adults with incomplete control. ACQ6 threshold changed from ≥1.5 to ≥0.8 during recruitment.	Three sessions (60 + 30 + 30 minutes) plus 10 minutes home practice twice daily; usual specialist care	6 and 12 months	Mini-AQLQ improved. ACQ6, Nijmegen and FEV _1_ between-group contrasts not significant. FeNO not an outcome.
Vagedes 2024 ^19^ RCT	60 Buteyko 30; usual treatment 30	German outpatient asthma population; ≥18 years; diagnostic guideline differed from GINA. Additional 30 healthy controls excluded here.	Five-day course, booster at one week, home practice twice daily for three months; usual treatment	3 months	Control pause, ACQ and Nijmegen improved. ICS contrast favoured Buteyko; beta-2 agonist contrast included no difference. No FEV _1_ or FeNO benefit.
Prem 2013 ^21^ Three-arm RCT	120 40 per arm	Indian outpatient asthma population	Buteyko or pranayama training for 3–5 days, then 15 minutes twice daily; pharmacological usual care	3 months	AQLQ improved versus usual care in both breathing arms; the Buteyko–pranayama contrast did not establish superiority of either technique. ACQ was also assessed.
Agarwal 2017 ^14^ Uncontrolled pre-post	60	India; stable asthma; mean age 25.45 years	Seven pranayama exercises for three months alongside existing therapy; no comparator	3 months	SGRQ improved within the cohort. Causal attribution is limited by lack of comparator.
Karam 2017 ^22^ Uncontrolled pre-post	74 enrolled 68 completed	USA immunotherapy clinic; persistent asthma; ages 18–65	Viloma pranayama, diaphragmatic and pursed-lip breathing; initial demonstration and brochure; twice daily; no comparator	1 month	ACT 19.7 to 21.3 (p = 0.002); Mini-AQLQ not significantly improved (p = 0.214). No inspiratory muscle training.
Evaristo 2020 ^29^ RCT	54 randomised 25 BG/29 AG analysed	Outpatient clinic, University Hospital, São Paulo, Brazil; moderate-to-severe persistent asthma; ages 30–65	Pranayama-based breathing exercises (24 sessions, 2/week, 40 min) vs aerobic treadmill training	3 months post-intervention	Similar ACQ6, AQLQ, HADS and airway-inflammation changes; aerobic arm 2.6× more likely to show clinical improvement at follow-up and a greater proportion achieving a > 5-day reduction in rescue-medication use (34% vs 8%; p = 0.04).
Ritz 2014 ^26^ RCT	120 randomised 61 CART/55 SLOW analysed	Academic/clinical research sites (SMU and Baylor University Medical Center), Dallas, TX, USA; adults with asthma of all severity grades; ages 18–65	Capnometry-assisted respiratory training (CART) vs slow breathing and awareness training (SLOW); 5 weekly sessions over 4 weeks plus home practice	1- and 6-month follow-up	Equal improvement in the primary asthma-control outcome (ACT, d = 0.81) and in SF-12 quality of life in both arms, with no significant between-group difference; CART showed greater increases in PCO _2_.
Coulson 2021/2022 ^30^ Quasi-randomised trial (alternation-based allocation)	90 allocated 45 exercise/45 control	Outpatient clinic, University of Michigan Medical Center, Ann Arbor, MI, USA; persistent asthma; age ≥ 65 years	Three-part breathing-exercise programme (viloma pranayama, diaphragmatic and pursed-lip breathing) vs an incentive-spirometer sham control, twice daily	1 month	Both groups improved on ACT and mini-AQLQ with no significant between-group difference; FEV _1_ declined similarly in both arms.
Erdoğan Yüce and Taşcı 2020 ^31^ RCT	55 randomised 25 pranayama/25 relaxation completed	Chest-disease outpatient clinics, public and private hospitals, Turkey; adults ≥18 years with chronic asthma ≥6 months	Pranayama breathing technique (20 min once daily for 1 month) vs progressive relaxation	1 month	Pranayama produced significantly higher ACT and AQLQ scores than relaxation (all p < 0.05); no significant between-group difference in PFT parameters or peak expiratory flow.
Rondinel 2014 ^32^ RCT	18 randomised 14 analysed (8 IS+EPAP/6 control)	Outpatient pulmonology clinic, Porto Alegre, Brazil; severe asthma; ages 20–80	Incentive spirometry combined with expiratory positive airway pressure, twice daily at home for 5 weeks; usual care	5 weeks	AQLQ and ACQ improved versus usual care (p = 0.02 and p = 0.002). No significant between-group difference in spirometry or 6-minute walk distance.
Sodhi 2014 ^33^ RCT	120 randomised allocation to groups not individually reported	Outpatient clinic and community yoga camps, Ludhiana, India; mild-to-moderate asthma; ages 17–50	Supervised yoga breathing weekly plus twice-daily home practice for 8 weeks; usual care	8 weeks	AQLQ domains and attack frequency/severity improved versus usual care (p < 0.05–0.01). No spirometry measured; participant flow and blinding not reported.
von Bonin 2018 ^34^ RCT (crossover)	56 enrolled 49 analysed, intention-to-treat	3 outpatient centres, Switzerland/Germany; not fully controlled asthma; ages ≥12 years (median 49)	Anthroposophic therapeutic speech, 11 weekly sessions plus home practice; waiting-list control phase	Median 182.5 days (both phases)	AQLQ and ACT improved versus the waiting-list phase (d = 0.86, p = 0.001; d = 0.53, p = 0.048). No spirometric change in either phase.
Chung 2021 ^35^ RCT	60 randomised 60 analysed (30 BTE/30 IMT)	Regional medical centre, Taiwan; moderate-to-severe asthma; ages 40–65	Conventional breathing exercises (BTE) vs inspiratory muscle training (IMT), both for 12 weeks; no usual-care arm	12 weeks	PImax improved in both arms, no significant between-group difference (p = 0.527). FVC increased with IMT but fell with BTE (interaction p = 0.045). ACT and ACQ improved similarly in both arms.
Kim 2024 ^36^ RCT	51 randomised 51 analysed, intention-to-treat (25 control/26 experimental)	University-hospital asthma clinic, São Paulo, Brazil; moderate-to-severe, not well-controlled asthma; ages 18–60	Aerobic exercise plus Buteyko breathing vs aerobic exercise plus muscle stretching, 20 sessions over 10 weeks	10 weeks	ACQ-7 and AQLQ improved within both groups, no significant between-group difference (p = 0.83 and p = 0.76). No significant between-group lung-function change.

**Table 4.** Corrected PEDro matrix for randomised and quasi-randomised trials. Y = criterion met; N = criterion not met. Items 2–11 contribute to the 0–10 score. Bruton, Andreasson, Prem, Evaristo, Ritz, Coulson, Erdoğan Yüce and Taşcı, Rondinel, Sodhi, von Bonin, Chung and Kim reproduce confirmed database ratings; Vagedes is a provisional editorial assessment. No PEDro total is assigned to uncontrolled studies.

Study	1	2	3	4	5	6	7	8	9	10	11	Total
Bruton	Y	Y	N	Y	N	N	Y	Y	Y	Y	Y	7
Andreasson	N	Y	N	Y	N	N	Y	Y	Y	Y	Y	7
Prem	Y	Y	Y	Y	N	N	Y	Y	N	Y	Y	7
Vagedes*	Y	Y	Y	N	N	N	N	Y	Y	Y	Y	6
Evaristo	Y	Y	Y	Y	N	N	Y	Y	Y	Y	Y	8
Ritz	Y	Y	N	Y	N	N	Y	Y	N	Y	Y	6
Coulson	Y	Y	N	Y	N	N	N	Y	Y	Y	Y	6
Erdoğan Yüce	Y	Y	Y	Y	N	N	Y	Y	Y	Y	Y	8
Rondinel	Y	Y	N	Y	N	N	Y	N	N	Y	Y	5
Sodhi	Y	Y	N	Y	N	N	N	N	N	Y	Y	4
von Bonin	Y	Y	N	Y	N	N	N	N	Y	Y	Y	5
Chung	Y	Y	Y	Y	N	N	N	Y	Y	Y	Y	7
Kim	Y	Y	Y	Y	N	N	Y	Y	Y	Y	Y	8

PEDro items: 1 eligibility; 2 random allocation; 3 concealed allocation; 4 baseline comparability; 5 participant blinding; 6 therapist blinding; 7 assessor blinding; 8 adequate follow-up (>85%); 9 intention-to-treat analysis; 10 between-group comparisons; 11 point estimates and variability. *Vagedes score requires author validation. The Andreasson item-1 rating reproduces the database entry and does not mean the source report lacks all eligibility information.

Confirmed ratings for the five further included trials accessed 13 October 2026: Rondinel https://search.pedro.org.au/search-results/record-detail/43001; Sodhi https://search.pedro.org.au/search-results/record-detail/38128; von Bonin https://search.pedro.org.au/search-results/record-detail/56648; Chung https://search.pedro.org.au/search-results/record-detail/64568; Kim https://search.pedro.org.au/search-results/record-detail/79737. Confirmed ratings accessed 10 October 2026: Bruton https://search.pedro.org.au/search-results/record-detail/52199; Andreasson https://search.pedro.org.au/search-results/record-detail/70501; Prem https://search.pedro.org.au/physiotherapy-choices/record-detail/32992; Evaristo https://search.pedro.org.au/search-results/record-detail/62040; Ritz https://search.pedro.org.au/search-results/record-detail/40705; Coulson https://search.pedro.org.au/search-results/record-detail/65466; Erdoğan Yüce and Taşcı https://search.pedro.org.au/search-results/record-detail/61023.

### Risk of bias assessment

Cochrane RoB 2 was formally completed, domain by domain, for all twelve included randomised trials against the review’s primary patient-reported outcome (asthma-related quality of life and/or asthma control), evaluated at each trial’s own pre-specified primary timepoint for that instrument where one was declared (6 months for Andreasson,14 the trial’s own designated primary timepoint despite a 12-month assessment also being reported; 6-month follow-up, 6MFU, for Ritz,26 the trial’s own sample-size/power target and its longest reported follow-up), or otherwise at the longest consistently reported follow-up for that instrument; the full domain-by-domain judgements are reported in Table 5, with signalling-question-level justification for every study and domain archived in the Zenodo data deposit.28 Patient-reported outcomes were obtained from participants who were necessarily aware of their allocation, because none of the included breathing-technique interventions can be concealed from the person receiving it; this structural feature, rather than the conduct of any single trial, is the principal driver of the Some concerns or High-risk domain judgements recorded across almost the entire included literature. Agarwal and Karam have no concurrent comparator, so RoB 2 is not applicable to either study; temporal changes, regression to the mean and nonspecific treatment effects cannot be separated from a breathing-exercise effect.

**Table 5.** Risk of bias (RoB 2) assessment of the twelve included randomised trials, by domain and overall judgement, for the review’s primary patient-reported outcome (asthma-related quality of life and/or asthma control), evaluated at each trial’s own pre-specified primary timepoint for that instrument where one was declared, or otherwise at the longest consistently reported follow-up (see Methods). Full signalling-question-level justification for every study and domain is archived in the Zenodo data deposit.28 Coulson is allocated by alternation rather than a concealed random sequence and is assessed descriptively rather than with RoB 2, consistent with Methods; Agarwal and Karam are uncontrolled pre-post designs with no concurrent comparator, for which RoB 2 is not applicable.

Study	D1: Randomisation process	D2: Deviations from intended interventions	D3: Missing outcome data	D4: Measurement of the outcome	D5: Selection of the reported result	Overall RoB 2 judgement
Bruton	Low	Low	Low	High	Low	High
Andreasson	Low	Low	Low	High	Low	High
Vagedes	Low	Low	Low	High	Some concerns	High
Prem	Low	High	High	High	Some concerns	High
Agarwal and Karam	N/A	N/A	N/A	N/A	N/A	Not applicable – uncontrolled pre-post design, no concurrent comparator
Evaristo	Low	Some concerns	Low	Some concerns	Some concerns	Some concerns
Ritz	Some concerns	Low	High	Some concerns	Some concerns	High
Coulson	Not assessed	Not assessed	Not assessed	Not assessed	Not assessed	Not assessed – allocation by alternation, not a concealed random sequence; assessed descriptively
Erdoğan Yüce and Taşcı	Low	Some concerns	Low	Some concerns	Some concerns	Some concerns
Rondinel	Some concerns	High	High	High	Some concerns	High
Sodhi	Some concerns	High	High	High	Some concerns	High
von Bonin	High	High	High	High	Some concerns	High
Chung	Some concerns	High	High	High	Some concerns	High
Kim	Some concerns	Low	Low	Some concerns	Some concerns	Some concerns

The four additionally included trials raise further considerations for risk of bias. Coulson30 is reclassified in this revision as quasi-randomised rather than randomised, because it allocated participants by alternation rather than a concealed random sequence; neither the investigators nor the outcome assessors were blinded, notwithstanding the trial’s own description as “blinded”. Erdoğan Yüce and Taşcı31 and Evaristo29 reported concealed allocation (sealed envelopes or a computer-generated sequence) with blinded outcome assessment. Ritz26 used block-randomised, computer-generated allocation but did not report whether allocation was concealed, and specified a protocol-driven withdrawal rule for participants who developed marked exacerbations (peak flow below 60% predicted with worsening symptoms) during the trial, a documented mechanism tying loss to follow-up directly to the trial’s own outcome-relevant measures. None of the four blinded participants or treatment providers, which is expected given the nature of a breathing-technique intervention. Coulson is allocated by alternation rather than a concealed random sequence and is therefore assessed descriptively rather than with RoB 2, consistent with the review’s methods; Erdoğan Yüce and Taşcı, Evaristo and Ritz have each now been formally assessed with Cochrane RoB 2 and are reported in Table 5; Ritz carries an overall High-risk judgement driven by this exacerbation-triggered withdrawal rule, a documented outcome-dependent missing-data mechanism, while Erdoğan Yüce and Taşcı and Evaristo retain an overall Some concerns judgement.

The five further included trials raise additional considerations for risk of bias. Rondinel32 used computer-generated randomisation without a reported allocation-concealment mechanism; the single outcome assessor was blinded to group allocation, but the analysis excluded one participant after an on-study exacerbation and was not described as intention-to-treat. Sodhi33 used permuted block randomisation but did not report allocation concealment, blinding of participants, therapists or outcome assessors, or any participant flow; the absence of a CONSORT-type flow diagram precludes assessment of attrition. Von Bonin34 reported that blinding of participants, therapists and treating clinicians was not feasible given the intervention, analysed results by intention-to-treat, and reported losses to follow-up (7 of 56 enrolled, 12.5%) concentrated in the active-treatment phase of its crossover design, described by the authors as mostly unrelated to the therapy (infection, a leg fracture); this is not the same as unrelated to the outcome, since the trial’s own diary instrument tracked infections alongside asthma exacerbations and symptom worsening, and no analysis specifically addressing this possibility was reported. Its own authors separately, explicitly reported trend and carry-over effects between the two crossover phases that, in their words, “hampered” their statistical analysis and reduced the design’s practical advantages; evaluated per Cochrane’s crossover-trial guidance, this unresolved carry-over, not the attrition pattern, is the basis for this trial’s Domain 2 High-risk judgement. Chung35 reported allocation by a blinded third party but explicitly stated in its own discussion that participants and outcome assessors were not blinded, and the same investigator delivered both interventions and performed outcome assessment; this is a substantive limitation not fully disclosed in the trial’s methods section. Kim36 was an open-label trial with participant and provider blinding not attempted, but outcome assessment and the statistical analysis were reported as blinded, and an intention-to-treat analysis was used. Rondinel, Sodhi, von Bonin, Chung and Kim have each now been formally assessed with Cochrane RoB 2 and are reported in Table 5; Rondinel, Sodhi, von Bonin and Chung carry an overall High-risk judgement, driven respectively by an imbalanced, outcome-relevant post-randomisation exclusion analysed without an intention-to-treat correction, by the absence of any reported blinding or participant-flow information, by unconcealed allocation combined with blinding not attempted at any level including the study statistician, and by an internal inconsistency between the trial’s stated methods and its own reported limitations regarding blinding.

### Health-related quality of life

The most precise comparative evidence for QoL comes from physiotherapy-based programmes in primary and specialist care. Bruton et al. ^13^ (n = 655) showed that AQLQ scores at 12 months favoured DVD-and-booklet (DVDB) delivery over usual care (adjusted mean difference 0.28; 95% CI 0.11–0.44; p = 0.002) and face-to-face delivery over usual care (0.24; 95% CI 0.04–0.44; p = 0.019). Andreasson et al. ^14^ (n = 193) found a Mini-AQLQ difference of 0.35 (95% CI 0.07–0.62; p = 0.015) at six months and 0.38 (95% CI 0.12–0.65) at twelve months. Both trials therefore support modest average improvements across different care settings. These mean contrasts were below the 0.5-point threshold commonly used for within-person clinically important change, although that threshold should not be directly equated with a between-group mean difference and individual responses varied.

Prem et al. ^21^ (n = 120) extends the QoL comparison to breathing techniques: at three months AQLQ favoured Buteyko over usual care by 0.97 (95% CI 0.48–1.46; p = 0.0001) and pranayama over usual care by 0.50 (95% CI 0.01–0.98; p = 0.042). The direct Buteyko–pranayama difference was 0.47 (95% CI −0.008 to 0.95; p = 0.056), which does not establish superiority of Buteyko. The distinct programmes and shorter follow-up prevent interpreting these estimates as a ranking against the physiotherapy trials.

Three of the five further included trials also compared a breathing-based intervention against a non-active comparator for quality of life. Rondinel ^32^ (n = 14 analysed) found a significantly larger improvement in global AQLQ with incentive spirometry combined with expiratory positive-airway pressure than with usual care after five weeks (median change 1.0 versus 0.3; p = 0.02), with 87.5% of the intervention group reaching the minimal clinically important difference versus 16.6% of controls. Sodhi ^33^ (n = 120) found significantly greater improvement in total AQLQ score with yoga-based breathing than with usual care at eight weeks (mean change from 127.60 to 142.65 versus 129.55 to 130.05; between-group p < 0.01), although the trial did not report a participant flow diagram, allocation concealment or blinding. Von Bonin ^34^ (n = 49 analysed, intention-to-treat), a crossover trial, found a significantly greater AQLQ improvement during the anthroposophic-therapeutic-speech phase than during the waiting-list phase (Cohen’s d = 0.86; p = 0.001), exceeding the instrument’s minimal clinically important difference; spirometric parameters did not improve in either phase.

Two further randomised trials and one quasi-randomised trial compared breathing exercises against an active, rather than a usual-care, comparator for quality of life. Erdoğan Yüce and Taşcı ^31^ (n = 50 completing) found significantly higher overall AQLQ and subscale scores for pranayama than for a progressive-relaxation control after one month (all p < 0.05), with large within-group improvements in both arms. Evaristo ^29^ (n = 54) found similar AQLQ gains for breathing exercises and aerobic training, with no significant between-group difference at any time point (group effect p = 0.909; group-by-time p = 0.468), although only the aerobic-training arm showed a significant within-group improvement on the related ACQ6 measure. Coulson ^30^ (n = 90) found no significant difference in mini-AQLQ change between a breathing-exercise programme and an incentive-spirometer sham control in adults aged 65 years or older (mean change 3.9 versus 5.9; p = 0.56). Ritz ^26^ (n = 116 analysed) compared two active breathing techniques and found similar improvement in general physical and mental health-related quality of life (SF-12) in both arms, with no significant between-group difference. Two of the five further included trials provide additional active-comparator evidence for quality of life. Kim ^36^ (n = 51, intention-to-treat) found similar within-group AQLQ improvement with aerobic exercise combined with Buteyko breathing and with aerobic exercise combined with muscle stretching (median change 0.56 versus 0.78), with no significant between-group difference (estimated effect 0.27; 95% CI −0.26 to 0.81). Chung ^35^ did not assess AQLQ. These comparative findings qualify the quality-of-life evidence: the benefit relative to usual care or a non-active control seen in Bruton, Andreasson, Prem, Rondinel, Sodhi and von Bonin is not matched by a consistent advantage over an active comparator or a sham control in the trials that used one.

Uncontrolled findings were mixed. Agarwal et al. ^20^ (n = 60) reported SGRQ total scores falling from 25.83 to 19.20 after three months (p < 0.001), with improvements in each domain. Karam et al. ^22^ (74 enrolled; 68 completing one month) found no statistically significant Mini-AQLQ improvement (p = 0.214). Thus QoL findings support a possible adjunctive benefit relative to usual care in several RCTs, but not a universal response across programmes or a consistent advantage over active comparators. The uncontrolled observations carry less causal weight and do not strengthen comparative efficacy estimates.

### Asthma control

Patient-reported asthma control was less consistent than QoL. In Vagedes et al., ^19^ Buteyko improved ACQ relative to usual treatment at three months (adjusted difference in change −0.48; 95% CI −0.88 to −0.09), with a reduction in Nijmegen Questionnaire scores. In contrast, Bruton ^13^ found no significant between-group ACQ or Nijmegen difference at twelve months, and Andreasson ^14^ found no significant six-month ACQ6 difference (−0.11; 95% CI −0.35 to 0.13) or Nijmegen difference. These results show that QoL gains do not necessarily coincide with measurable improvement in asthma control or hyperventilation-related symptoms. Among the five further included trials, Rondinel ^32^ found a significantly larger ACQ improvement with incentive spirometry and expiratory positive-airway pressure than with usual care at five weeks (median change −1.0 versus 0.1; p = 0.002; minimal clinically important improvement in 100% versus 0% of participants). von Bonin, ^34^ using a crossover design against a non-active waiting-list phase, found a significant ACT improvement by intention-to-treat analysis (Cohen’s d = 0.53; p = 0.048), equivalent to a 2.09-point between-phase difference below the instrument’s three-point minimal clinically important difference; this ACT effect was no longer significant in the trial’s per-protocol analysis (p = 0.121).

Prem ^21^ reported an ACQ reduction of 0.44 within the Buteyko group (95% CI 0.23–0.64; p = 0.001), whereas the pranayama and control changes were nonsignificant. These are within-group contrasts and do not themselves establish a between-group effect. The ACT and pranayama-specific breathlessness claims in the previous manuscript were unsupported and have been removed. In Karam, ^22^ ACT rose from 19.7 to 21.3 at one month (p = 0.002), but the uncontrolled design precludes attributing this change to treatment. Differences in sample, comparator, instrument and follow-up limit cross-study comparisons.

Andreasson ^14^ also reported a secondary improvement in the HADS depression subscale at six months (difference − 0.90; 95% CI −1.67 to −0.14). This is a distinct psychological outcome, not evidence of improved ACQ or of a dose-response effect. The trial prescribed home practice twice daily and did not test the previously asserted comparison of three versus two daily sessions.

The four additionally included trials add further, mixed comparative evidence on asthma control. Erdoğan Yüce and Taşcı ^31^ found significantly higher ACT scores for pranayama than for relaxation after one month (21.84 versus 18.68; between-group p = 0.021), the clearest comparative advantage for an active breathing technique over an active control identified in this review. Ritz ^26^ found similarly large within-group ACT improvement in both the capnometry-assisted and slow-breathing arms (d = 0.81) with no significant group-by-time interaction (p = 0.157), indicating comparable benefit from either breathing technique rather than an advantage specific to one. Coulson ^30^ found no significant between-group difference in ACT change in older adults (1.3 versus 1.02; p = 0.78). Evaristo ^29^ found a significant between-group difference favouring aerobic training: participants in the aerobic arm were 2.6 times more likely to show clinically important ACQ6 improvement than those in the breathing-exercise arm at three months (RR 2.6; 95% CI 1.2–10; p = 0.02), although the continuous between-group ACQ6 difference was not statistically significant (adjusted difference − 0.38; 95% CI −0.86 to 0.11; p = 0.13 at three months). Chung ^35^ and Kim ^36^ provide further active-comparator evidence restricted to asthma-control instruments other than AQLQ. Chung ^35^ found significant within-group ACT and ACQ improvement over 12 weeks in both the conventional-breathing-exercise and inspiratory-muscle-training arms, with no significant between-group difference for either instrument (ACT p = 0.927; ACQ p = 0.883); the authors noted a possible ceiling effect, as baseline scores already indicated reasonably controlled asthma. Kim ^36^ similarly found significant within-group ACQ-7 improvement with aerobic exercise combined with Buteyko breathing and with aerobic exercise combined with muscle stretching, with no significant between-group difference (p = 0.83); both groups reached the ACQ-7 minimal clinically important difference. Taken together with Vagedes, Bruton, Andreasson, Rondinel and von Bonin, asthma-control results remain inconsistent across programmes, comparators and populations, with the clearest comparative benefit for breathing exercises seen only against a non-breathing relaxation control in a single trial.

### Spirometric and physiological parameters

The selected RCTs did not establish a consistent improvement in spirometric indices. Bruton ^13^ and Andreasson ^14^ found no significant between-group FEV _1_ benefit. FeNO was reported without significant intervention effects in Bruton ^13^ and Vagedes ^19^; it was not reported as an outcome in Andreasson. Absence of a detected FeNO effect in these trials does not establish that every inflammatory pathway is unaffected or identify the mechanism of benefit.

Vagedes ^19^ found an increase in control-pause duration and a small change in threshold dead-space volume (adjusted difference in change 11.0 mL; 95% CI 0.01–21.9). These measures differ from FEV _1_ and patient-reported control. The trial does not substantiate the previous claim of a demonstrated increase in end-tidal CO _2_ or restoration of carbon dioxide levels as the causal mechanism.

The four additionally included trials likewise found no consistent spirometric benefit. Coulson ^30^ found a similar decline in percent-predicted FEV _1_ in both arms (−6.5% versus −7.3%; p = 0.82). Erdoğan Yüce and Taşcı ^31^ found no significant between-group difference in FEV _1_, FVC or FEV _1_/FVC, and the increase in peak expiratory flow was significant only within the pranayama group, not between groups (p = 0.080). Ritz ^26^ found FEV _1_ unchanged in both arms, while end-tidal PCO _2_ increased more with capnometry-assisted training than with slow breathing (d = 1.45 versus 0.64); this PCO _2_-specific effect does not extend to spirometric outcomes.

The five further included trials add mixed evidence on spirometric and respiratory-muscle parameters, without establishing a consistent FEV _1_ benefit. Rondinel ^32^ found no significant between-group difference in spirometric variables after five weeks. Sodhi ^33^ did not measure spirometry or objective lung function. Von Bonin ^34^ found no significant change in FEV _1_, FVC or FEV _1_/FVC in either the intention-to-treat or per-protocol analysis. Chung ^35^ found no significant between-group difference in FEV _1_ (p = 0.505), but a significant interaction for FVC (p = 0.045), driven by an increase with inspiratory muscle training (+4.25% predicted) against a decrease with conventional breathing exercises (−1.43% predicted); the FVC change in the training arm did not reach the minimal clinically important difference. Both arms in Chung ^35^ showed a significant increase in maximal inspiratory pressure (PImax) over time (16.92% with conventional exercises and 29.84% with inspiratory muscle training), with no significant between-group difference (p = 0.527). Kim ^36^ reported no significant improvement in lung-function variables in either group, including between-group differences, although exact values were reported only in supplementary material not available for independent verification here.

### Rescue medication use and healthcare resource utilisation

Medication findings were limited and depended on the analysis. In Vagedes, ^19^ beta-2 agonist use decreased within the Buteyko group, but its adjusted between-group contrast was −68.6 μg/day salbutamol equivalents (95% CI −144.6 to 7.5), which includes no difference. The ICS contrast favoured Buteyko (−245.5 μg/day beclometasone equivalents; 95% CI −463.1 to −27.9). These findings from one small trial do not establish a general medication step-down strategy. In Karam, ^22^ 66.1% of respondents perceived reduced rescue-inhaler use; this was an uncontrolled survey response, not an objectively quantified comparative dose reduction. Sodhi ^33^ recorded a weekly medication-dosage diary and found no significant between-group difference at eight weeks (mean dosage 2.48 versus 2.82; p > 0.10), although a significant within-group reduction was observed in the yoga arm alone. None of the other four further included trials reported rescue-medication use or healthcare utilisation as a distinct outcome.

Evaristo ^29^ found that a greater proportion of participants in the aerobic-training arm than in the breathing-exercise arm achieved a clinically meaningful (more than 5-day) reduction in the number of days with rescue-medication use (34% versus 8%; RR 4.3; 95% CI 1.04–17.8; p = 0.04; NNT = 4), a further instance in which an active comparator outperformed breathing exercises rather than the reverse.

Bruton ^13^ found no significant reduction in primary-care consultations or asthma attacks. Its economic analysis nevertheless reported numerically lower mean annual medical costs for DVDB (£296) and face-to-face retraining (£335) than usual care (£356), with uncertain cost differences and a favourable cost-effectiveness probability for the inexpensive DVDB programme. Equivalence between the two delivery arms was demonstrated for AQLQ within a prespecified ±0.3-point margin (difference 0.04; 95% CI −0.16 to 0.24). This supports those two delivery formats for that programme and outcome, without establishing equivalence of all digital interventions or prevention of exacerbations.

### Safety

The larger trials did not suggest an increased adverse-event burden: Andreasson ^14^ reported adverse events in 14.9% versus 18.1% (p = 0.38), and Bruton ^13^ reported events, including serious events, in all groups. In Vagedes, ^19^ one usual-treatment participant was hospitalised for sinusitis and otitis media and no other adverse events were reported. Smaller and uncontrolled reports offered limited systematic safety information. Overall, available reporting is reassuring but insufficient to exclude uncommon harms or establish safety across all techniques and severities. Evidence on exacerbations is also insufficient for a preventive claim; nonsignificant findings do not prove absence of an effect.

Among the four additionally included trials, Evaristo ^29^ reported asthma exacerbations as a non-study-related adverse event in 35% of the aerobic-training arm and 24% of the breathing-exercise arm, with no significant between-group difference (p = 0.31); Ritz ^26^ and Coulson ^30^ reported no study-related adverse events in either arm. These additional findings are consistent with the broader conclusion that available safety reporting is reassuring but remains insufficient to exclude uncommon harms.

Safety reporting among the five further included trials was similarly limited and, in two cases, better documented than in the originally selected studies. Rondinel ^32^ reported one protocol-related complication in the intervention group, leading to exclusion of that participant from the analysis after an asthma exacerbation and hospitalisation; no adverse events were reported in the control group. Sodhi ^33^ and Chung ^35^ reported no adverse-event information at all. Von Bonin ^34^ reported no serious adverse events, but 15 participants described 22 minor difficulties (mainly practising the exercises at home) and therapists recorded 20 minor adverse events in 14 participants (most commonly cough or throat irritation) across 539 individual sessions. Kim ^36^ reported that some participants found the Buteyko technique difficult in the first weeks, with one exacerbation and one case of worsening rhinitis during training, after which exercises resumed without further pausing; no comparable difficulties were reported in the active-comparator arm. These findings do not suggest an increased serious-adverse-event burden with any of the five further interventions, but reporting remains too limited and heterogeneous to exclude uncommon harms.

## Discussion

Across the fifteen included studies, QoL is the outcome with the clearest comparative support, whereas asthma control, Nijmegen scores, medication use and physiological outcomes show a less uniform pattern. This distinction is clinically relevant: an intervention can improve the experience of living with asthma without producing the same change in ACQ, FEV _1_ or exacerbations. Conclusions should therefore be outcome-specific and should give greater weight to comparative trial results than to uncontrolled pre-post improvement.

These findings are broadly compatible with the cautious conclusions of Santino et al., ^12^ whose review already included the BREATHE trial and rated outcome-specific certainty from moderate to very low. Andreasson ^14^ adds evidence from specialist care after that search period, and Vagedes ^19^ adds a later Buteyko trial. The overview by Liu et al. ^23^ was published in July 2025, after the March 2025 search end date; it can inform discussion but cannot be represented as a report excluded during that original search. Our synthesis has no formal GRADE assessment, so we do not assign moderate or high certainty to its findings.

Clinical heterogeneity includes asthma severity and baseline control, primary versus specialist care, breathing-exercise components, supervised contact, home-practice duration and follow-up. Methodological differences include usual-care versus active comparators (the active comparators across the included trials span aerobic exercise training, progressive relaxation, a sham-spirometer device, an alternative breathing technique and, among the five further included trials, inspiratory muscle training and a combined aerobic-and-breathing programme), three-arm designs versus two-arm designs, uncontrolled observations, outcome instruments and awareness of allocation. These differences may affect both effect magnitude and susceptibility to expectation or attention effects. They justify separating evidence by outcome and design; they do not by themselves demonstrate statistical heterogeneity or prove that comparable subsets could never be pooled.

Changes in symptom perception, relaxation, breathing patterns or confidence in self-management are plausible explanations for QoL gains, but the trials do not establish a single causal mechanism. ^6^ ^,^ ^7^ ^,^ ^24^ ^,^ ^25^ Persistent symptoms and features of dysfunctional breathing may prompt consideration of retraining alongside appropriate assessment, but a Nijmegen score alone is not a validated predictor of treatment response in this synthesis.

Bruton ^13^ supports the potential accessibility of self-guided DVD-and-booklet retraining when the tested programme is offered to a similar population. Its equivalence result cannot be extended to all current applications or remote programmes. Differences in training intensity and adherence across studies also do not establish an optimal dose. No randomised dose comparison supporting three daily sessions over two was identified in Andreasson.

The small Vagedes trial ^19^ suggests that medication use merits further investigation, but nonsignificant between-group beta-2 agonist findings and limited long-term follow-up temper interpretation. Prem ^21^ did not demonstrate statistically significant superiority of Buteyko over pranayama for QoL. The present evidence therefore does not justify ranking Buteyko as the most effective technique or using breathing exercises as the basis for automatic corticosteroid reduction.

Evidence on exacerbations and serious harms remains limited. GINA ^1^ positions breathing exercises as a supplement to pharmacotherapy. Any improvement in self-reported symptoms should be interpreted alongside objective assessment and ongoing guideline-based treatment; it is not evidence that anti-inflammatory therapy is unnecessary.

Review-process limitations remain substantial despite the corrections made in this revision. The original search strategies, exact dates, record-level selection decisions and amendment history cannot be reconstructed from the retained material, and the PRISMA flow diagram has not been rebuilt with validated denominators. Four previously unresolved candidate reports (26, 29, 30 and 31) and five further candidate reports identified by an updated PubMed/MEDLINE search for this revision (32, 33, 34, 35 and 36) have now been checked against the review’s own eligibility criteria using their full texts and incorporated as included studies; this resolves their specific status but does not confirm that the original search and screening were complete, nor does it rule out other eligible reports that may remain unidentified. Notably, the updated search did not retrieve Ritz,26 an already-included study, indicating that even a reproducible database search using the review’s own core expression may not capture every eligible report; this sensitivity limitation is disclosed rather than resolved. The age exception in Bruton, the inclusion of participants from 17 years in Sodhi and from 12 years in von Bonin, the restriction to participants aged 65 years or older in Coulson, and variation in diagnostic definitions further limit applicability to a strictly adult, uniformly confirmed asthma population. The review was not prospectively registered; uncontrolled studies were added without a verifiable amendment record; no formal certainty assessment, statistical heterogeneity analysis or sensitivity analysis was undertaken; and outcome-specific risk-of-bias (RoB 2) signalling-question judgements, now completed for the twelve included randomised trials ( Table 5), have not been attempted for Coulson, which is quasi-randomised, nor are they applicable to Agarwal and Karam, which are uncontrolled pre-post designs. The one- to twelve-month follow-up, limited safety reporting and 2013 date restriction also constrain interpretation. Reporting and publication bias cannot be excluded.18 The present revision corrects verifiable source-level errors and incorporates the nine additionally confirmed studies identified across both targeted audits, but it does not reconstruct the undocumented original screening log or the full PRISMA flow.

## Conclusions

Structured breathing retraining may improve health-related QoL as an adjunct to pharmacological care, relative to usual care or a non-active control in six trials. Against active comparators the picture is more mixed: one trial favoured breathing exercises over progressive relaxation, three trials found no significant difference against aerobic training, a sham incentive-spirometer control or combined aerobic exercise, and two trials found no statistically significant between-group difference between two active breathing-based techniques. Evidence that breathing retraining improves patient-reported asthma control is similarly mixed, and superiority of one technique has not been established. The heterogeneity of programmes, populations, comparators and designs limits generalisability; uncontrolled observations should be considered exploratory.

These fifteen included studies do not establish consistent lung-function improvement, a mechanism independent of every inflammatory pathway, or prevention of severe exacerbations, and do not support a general reduction in rescue medication: indeed, one trial found a larger reduction in rescue-medication use with aerobic training than with breathing exercises. Breathing exercises may be considered within multidisciplinary care for persistent symptoms, while retaining guideline-based pharmacological treatment. Confidence in the review-level conclusion is additionally limited by the consistently elevated risk of bias found across the twelve randomised trials ( Table 5) and by original search and selection documentation that could not be reconstructed.

Future work should use prospectively registered eligibility criteria, reproducible database searches, outcome-specific bias assessment and formal certainty appraisal. Trials should compare defined techniques and practice doses, assess predictors of response and adherence, and evaluate exacerbations, safety and costs over longer follow-up. For this review, the eligibility of the previously unresolved candidate reports identified in two successive targeted audits, including an updated PubMed/MEDLINE search, has now been checked against the full texts and incorporated, and formal outcome-specific risk-of-bias assessment has been completed for the twelve included randomised trials; reconciliation of the original selection flow remains necessary before a definitive final synthesis.

## Author contributions

**Astrid Crespo-Lessmann** : Conceptualization, Methodology, Investigation, Writing – Original Draft Preparation, Writing – Review & Editing, Project Administration. **Eduardo Vélez-Segovia **: Investigation, Formal Analysis, Writing – Review & Editing. Luis Paz: Investigation, Data Curation, Writing – Review & Editing. **Vicente Plaza**: Conceptualization, Supervision, Validation, Writing – Review & Editing.

## Artificial Intelligence Disclosure

The original manuscript disclosed use of Claude (claude-sonnet-4-6, Anthropic) for writing assistance. Claude also assisted with this revision, including language editing and source checking, but did not perform a repeat systematic search or new statistical analysis. The authors are responsible for verifying all extracted values, eligibility decisions, methodological assessments and final statements before submission.

## Data Availability

The published version links the underlying and extended review materials to Zenodo (doi: 10.5281/zenodo.20499963). ^28^ These comprise the extraction file, methodological-assessment file, full-text exclusion summary and PRISMA checklist. The corrected copies prepared for this revision identify verified corrections and unresolved audit issues, and now cover all fifteen included studies following confirmation of eligibility for the four previously unresolved candidate reports (Evaristo, Ritz, Coulson, and Erdoğan Yüce and Taşcı) and the five further candidate reports identified by an updated PubMed/MEDLINE search for this revision (Rondinel, Sodhi, von Bonin, Chung and Kim). These corrected files have now been deposited as an updated public version (doi: 10.5281/zenodo.23556744), and the repository reference has been updated accordingly. Primary study data remain those of the original publications. The revised PRISMA checklist explicitly distinguishes reported information from incomplete or unavailable items. Table 2 reproduces the historical reported selection counts with audit notes, rather than claiming a reconstructed final PRISMA diagram. Database-specific original search exports and the record-level screening log are unavailable. Methodological documentation for the updated PubMed/MEDLINE search conducted for this revision (search string, date, and screened and retrieved record counts) is retained with the underlying review materials.
