# Supplementary material for: Breathing Exercises as Adjunct Therapy in Adult Asthma: A Systematic Review
Source: F1000Res. 2026 Oct 31;15:996. Originally published 2026 Jun 23. [Version 2] doi: 10.12688/f1000research.180230.2 (PMC13714416; doi:10.12688/f1000research.180230.2)
Supplement: Peer review history

# Reviewer report: Reviewer response for version 1

**DOI:** 10.5256/f1000research.198820.r524394

**Published:** 2026-10-27

**Authors:** Mariana Muñoz

**Affiliations:** 1 Bellvitge University Hospital, Catalunya, Spain

General Comments

 The systematic review by Crespo et al. aims to summarize the available evidence on structured breathing exercise programs as an adjunctive therapy in adults with asthma. The authors conducted a literature search covering the period from January 2013 to March 2025 and identified six studies that met the predefined inclusion criteria.

 The topic is clinically relevant, given the potential role of non-pharmacological interventions in asthma management. However, several aspects of the manuscript require further consideration, particularly regarding the presentation and interpretation of the results and the strength of the conclusions.

 Major Comments

**1. Study objectives and interpretation of the evidence**

 The authors state that the objective of the review is to evaluate the efficacy and safety of structured breathing exercise programs. However, given the characteristics and limitations of the included studies, the manuscript primarily provides a descriptive synthesis of previously published findings rather than a conclusive assessment of the efficacy and safety of these interventions.

 I recommend ensuring greater consistency between the stated objectives, the results presented, and the conclusions drawn. In particular, the conclusions should be interpreted cautiously, definitive statements that are not sufficiently supported by the available evidence.

**2. Presentation of the results**

 I suggest revising the structure and presentation of the Results section. Currently, the findings are mainly presented as a sequential description of individual studies and their respective outcomes, making it difficult to obtain an integrated overview of the evidence. A more cohesive narrative, organized according to relevant clinical outcomes or key findings, would improve readability and facilitate comparisons across studies. Detailed information on individual studies could be presented in the corresponding tables, avoiding unnecessary repetition in the main text.

**3. Characteristics of the study populations**

 In the Discussion section, the authors acknowledge the importance of the characteristics of the populations receiving these interventions. However, the clinical characteristics of the participants included in the six studies are not sufficiently discussed. I recommend providing a more detailed description of the study populations, as differences in baseline characteristics may influence the response to breathing exercise interventions and the generalizability of the findings.

 In particular, Table 3 could be expanded to include information on baseline asthma control, such as the proportion of participants with controlled, partly controlled, or uncontrolled asthma, whenever these data are available. This would help contextualize the findings and assess their applicability to different asthma populations.

**4. Conclusions**

 The Conclusions section is excessively long and includes statements that appear too definitive considering the limited available evidence. I recommend shortening this section and focusing on the main findings of the review, while acknowledging the uncertainty and limitations of the evidence.

 Discussion of potential underlying mechanisms and future research directions would be more appropriately addressed in the Discussion section rather than in the Conclusions.

 Minor Comments

**1. Abbreviations and acronyms**

 The authors should carefully review the use of abbreviations and acronyms throughout the manuscript and tables. Some abbreviations are not properly defined when first introduced in the text or are missing from the table footnotes.

Are the rationale for, and objectives of, the Systematic Review clearly stated?

Yes

Is the statistical analysis and its interpretation appropriate?

Not applicable

If this is a Living Systematic Review, is the ‘living’ method appropriate and is the search schedule clearly defined and justified? (‘Living Systematic Review’ or a variation of this term should be included in the title.)

Partly

Are sufficient details of the methods and analysis provided to allow replication by others?

Yes

Are the conclusions drawn adequately supported by the results presented in the review?

Yes

Reviewer Expertise:

Clinical research in severe asthma

I confirm that I have read this submission and believe that I have an appropriate level of expertise to confirm that it is of an acceptable scientific standard, however I have significant reservations, as outlined above.

# Reviewer report: Reviewer response for version 1

**DOI:** 10.5256/f1000research.198820.r524395

**Published:** 2026-10-08

**Authors:** David Clofent

**Affiliations:** 1 Vall d'Hebron University Hospital, Barcelona, Spain

This manuscript presents a systematic review evaluating the efficacy and safety of structured breathing exercises as an adjunctive intervention in adults with asthma. The authors searched five databases for randomised controlled trials, controlled quasi-experimental studies, and uncontrolled pre-post studies published between 2013 and 2025. The authors conclude that breathing exercises may be a useful adjunct to pharmacological treatment, particularly in patients with persistent symptoms and features of dysfunctional breathing.

 Overall, this is a clinically relevant and clearly structured systematic review addressing an important non-pharmacological intervention in adult asthma. The inclusion of two large, contemporary randomised controlled trials strengthens the clinical relevance of the review, particularly regarding breathing retraining in patients with persistent symptoms and the potential role of alternative delivery formats.

 I only have a few minor suggestions:

 - Methods: The sentence “studies scoring ≥6 ...” is grammatically incomplete. Please revise to:

 “studies scoring ≥6 were...”

 Results: The sentence “Bruton et al. (n = 655) showed AQLQ…” is missing the conjunction *that*. Please revise to: “Bruton et al. (n = 655) showed that AQLQ scores…”

 Conclusions: consider replacing the expression “reduces asthma control symptom scores” with 

 “improves patient-reported asthma control”.

 As an additional comment, please clarify when and why the eligibility criteria were broadened to include uncontrolled pre-post studies. In particular, it would be helpful to know whether this amendment was made before or after study screening began and how this change affected the review process. This is mainly a request for clarification and does not necessarily need to be addressed in the manuscript.

Are the rationale for, and objectives of, the Systematic Review clearly stated?

Yes

Is the statistical analysis and its interpretation appropriate?

Yes

If this is a Living Systematic Review, is the ‘living’ method appropriate and is the search schedule clearly defined and justified? (‘Living Systematic Review’ or a variation of this term should be included in the title.)

Yes

Are sufficient details of the methods and analysis provided to allow replication by others?

Yes

Are the conclusions drawn adequately supported by the results presented in the review?

Yes

Reviewer Expertise:

Pulmonology, Respiratory Diseases, Interventional Pulmonology, Airway Disease

I confirm that I have read this submission and believe that I have an appropriate level of expertise to confirm that it is of an acceptable scientific standard.

# Response

**Published:** 2026-10-22

**Authors:** Astrid Crespo-Lessmann

**Affiliations:** Respiratory and Allergy, Hospital de la Santa Creu i Sant Pau, Barcelona, Catalonia, Spain

Dear Dr. Clofent,

 Thank you for your helpful comments and positive assessment. We have addressed the language corrections and revised the explanation of the eligibility amendment as follows.

 1 Methods wording

 The incomplete sentence has been corrected to “studies scoring ≥6 were classified as good quality”. The same paragraph now specifies that PEDro items 2–11, rather than 2–10, contribute to the 0–10 score, and distinguishes confirmed database ratings from the provisional source-based appraisal.

 Location: Methods — Quality assessment; Table 4.

 2 Results wording

 The sentence now reads “Bruton et al. (n=655) showed that AQLQ scores at 12 months…” and appears within a cross-study synthesis of quality-of-life findings.

 Location: Results — Health-related quality of life.

 3 Conclusions wording

 We have replaced the expression about reducing asthma-control symptom scores with wording about improving patient-reported asthma control. The full statement is deliberately qualified: “Evidence that it improves patient-reported asthma control is less consistent”, because Bruton and Andreasson did not find significant between-group ACQ differences. The Abstract and Discussion use the same interpretation.

 Locations: Abstract; Discussion; Conclusions.

 4 Timing and rationale for broadening eligibility

 We agree that this requires a precise account. The available material shows that uncontrolled pre-post studies were included after a change from a stricter design criterion, but contains no dated protocol amendment or record-level screening log. We therefore cannot establish whether this decision preceded screening, occurred during title/abstract screening, or followed full-text review. We also cannot verify whether previously excluded records were rescreened, searches were repeated without design filters, or both reviewers participated in such reassessment.

 Accordingly, we withdraw an unsubstantiated explanation that had located the amendment after title/abstract and full-text screening because “only four RCTs” were found. That account cannot be substantiated and should not be offered as fact. The revised Methods and Discussion state that the timing and process are undocumented and acknowledge the associated selection-bias concern. Uncontrolled findings are presented separately as exploratory observations and are not used to establish comparative efficacy. Their possible contribution is feasibility and acceptability information, but this interpretive use is not presented as a verified contemporaneous reason for the amendment.

 Locations: Methods — Protocol and registration; Eligibility criteria; Study selection; Results — Health-related quality of life and Asthma control; Discussion — review-process limitations.

 Our present source audit also identified four candidate reports that had been absent from the original selection without a documented reason (Evaristo 2020, Ritz 2014, Coulson 2021/2022, and Erdoğan Yüce and Taşcı 2020). We have since checked each against the review's eligibility criteria using its full text and confirmed all four eligible; they are now included. For this revision we further conducted an updated, dated and reproducible PubMed/MEDLINE search (12 October 2026, within the original January 2013–March 2025 window), which identified five additional eligible randomised trials not present in the original selection (Rondinel 2014/2015, Sodhi 2014, von Bonin 2018, Chung 2021, and Kim 2024); these were likewise confirmed eligible using their full texts and added, bringing the total to fifteen studies and 1,786 enrolled/randomised participants, with their results incorporated throughout the Results, Discussion, Conclusions and Tables 3–5. Notably, this updated search did not retrieve Ritz, an already-included study, indicating a possible sensitivity limitation of the original search strategy; this is disclosed rather than resolved. This targeted check does not recreate the original search and screening process, so we cannot confirm that no further eligible report remains unidentified; this is disclosed explicitly as an open limitation rather than presented as resolved. Formal outcome-specific risk-of-bias assessment (Cochrane RoB 2) is now complete for all twelve included randomised trials and is reported in Table 5; it does not apply to Coulson (quasi-randomised) or to Agarwal and Karam (uncontrolled).

 Thank you again for your careful review.

 Astrid Crespo-Lessmann,

 on behalf of all authors

# Reviewer report: Reviewer response for version 1

**DOI:** 10.5256/f1000research.198820.r510755

**Published:** 2026-08-31

**Authors:** Lu Shi

**Affiliations:** 1 Tufts Medical Center, Boston, Massachusetts, USA

Overall, this is a well-written paper. However, I would suggest that the authors restructure the Results section to read more cohesively. At present, the results of each included study are reported separately, without exploring potential connections across studies. 

 Because the Results section does not present the findings cohesively, the rationale for this study is not clear to me. I would suggest that the authors better articulate the importance and rationale of the work, rather than conducting a review for its own sake. 

 In the Discussion, we would expect the authors to address the heterogeneity across the included studies. Based on the result, I would suggest the author make a more conservative conclusion.

Are the rationale for, and objectives of, the Systematic Review clearly stated?

Partly

Is the statistical analysis and its interpretation appropriate?

Yes

If this is a Living Systematic Review, is the ‘living’ method appropriate and is the search schedule clearly defined and justified? (‘Living Systematic Review’ or a variation of this term should be included in the title.)

Not applicable

Are sufficient details of the methods and analysis provided to allow replication by others?

Yes

Are the conclusions drawn adequately supported by the results presented in the review?

Partly

Reviewer Expertise:

asthma, clincial trial

I confirm that I have read this submission and believe that I have an appropriate level of expertise to confirm that it is of an acceptable scientific standard, however I have significant reservations, as outlined above.

# Response

**Published:** 2026-10-22

**Authors:** Astrid Crespo-Lessmann

**Affiliations:** Respiratory and Allergy, Hospital de la Santa Creu i Sant Pau, Barcelona, Catalonia, Spain

Dear Dr. Shi,

 Thank you for your constructive assessment. We agree that a cohesive synthesis and more cautious interpretation are needed. The revised text addresses your points as follows.

 1 Results structure

 We have rewritten the Results as a synthesis by outcome and study design, rather than a sequence of isolated study summaries. The quality-of-life subsection now compares the direction, magnitude and precision of the findings from Bruton, Andreasson and Prem, and then discusses Agarwal and Karam separately as uncontrolled evidence. The asthma-control subsection explicitly contrasts the positive ACQ finding in Vagedes with the nonsignificant between-group findings in Bruton and Andreasson. These changes show that quality-of-life improvement is more consistently supported than improvement in patient-reported asthma control.

 Locations: Results — Health-related quality of life; Asthma control; Spirometric and physiological parameters; Rescue medication use and healthcare resource utilisation; Safety; Tables 3–5.

 2 Importance and rationale

 The Introduction now explains the clinical question: whether breathing retraining adds patient-important benefit to pharmacological care, whether gains in quality of life coincide with better control or physiological outcomes, and how setting and delivery influence interpretation. We have corrected an inaccurate statement: Santino et al. (2020) already included the BREATHE trial reported by Bruton, under the identifier Thomas 2017; it is not new evidence relative to that review. Later trials, particularly Andreasson (2022) and Vagedes (2024), provide the rationale for reassessing this evidence. We also corrected the description of Cochrane certainty to moderate to very low, depending on outcome.

 Locations: Introduction — Why this review is important; Objectives; Discussion.

 3 Heterogeneity and strength of conclusions

 The Discussion now addresses variation in asthma severity and baseline control, care setting, intervention components, supervision, practice requirements, comparators, outcome instruments, study design and follow-up. We distinguish these clinical and methodological differences from statistical heterogeneity, which was not quantified. The Conclusions now focus on a possible adjunctive quality-of-life benefit and acknowledge mixed asthma-control findings. We have removed claims of established Buteyko superiority, a dose-response effect, a proven anti-inflammatory mechanism, general medication reduction, universal safety or prevention of exacerbations. Equivalence of DVD-and-booklet and face-to-face delivery is limited to the programme and AQLQ comparison tested by Bruton.

 Locations: Methods — Data synthesis; Discussion; Conclusions; Abstract.

 Additional source and process corrections

 Source checking identified further errors: Karam enrolled 74 participants (68 completed), not 10; its Mini-AQLQ change was nonsignificant. We also completed a targeted eligibility check of four candidate reports that had been absent from the original selection without a documented reason (Evaristo 2020, Ritz 2014, Coulson 2021/2022, and Erdoğan Yüce and Taşcı 2020); all four were confirmed eligible against the review's own criteria using their full texts and have been added as included studies. For this revision we further conducted an updated, dated and reproducible PubMed/MEDLINE search (12 October 2026, within the original January 2013–March 2025 window), which identified five additional eligible randomised trials not present in the original selection (Rondinel 2014/2015, Sodhi 2014, von Bonin 2018, Chung 2021, and Kim 2024); these were likewise confirmed eligible using their full texts and added, bringing the total to fifteen studies and 1,786 enrolled/randomised participants. Notably, this updated search did not retrieve Ritz, an already-included study, indicating a possible sensitivity limitation of the original search strategy; this is disclosed rather than resolved. We corrected the extraction and PEDro tables accordingly and incorporated all nine additionally confirmed studies' results throughout the Results, Discussion, Conclusions and Tables 3–5. Formal outcome-specific risk-of-bias assessment (Cochrane RoB 2, with signalling questions) is now complete for all twelve included randomised trials and is reported in Table 5; it does not apply to Coulson (quasi-randomised) or to Agarwal and Karam (uncontrolled). One issue remains genuinely open and is disclosed as a limitation rather than presented as resolved: the original search and title/abstract screening cannot be reconstructed from the retained material, so we cannot confirm that no further eligible report remains unidentified.

 Thank you for helping us improve the coherence and restraint of the manuscript.

 Astrid Crespo-Lessmann,

 on behalf of all authors
